# Moment estimators of relatedness from low-depth whole-genome sequencing data

**DOI:** 10.1186/s12859-022-04795-8

**Published:** 2022-06-24

**Authors:** Anthony F. Herzig, M. Ciullo, FranceGenRef Consortium, Jean-François Deleuze, Emmanuelle Génin, Richard Redon, Chantal Adjou, Stéphanie Chatel, Claude Férec, Marcel Goldberg, Philippe-Antoine Halbout, Hervé Le Marec, David L’Helgouach, Karen Rouault, Jean-Jacques Schott, Anne Vogelsperger, Marie Zins, Delphine Bacq, Hélène Blanchet, Anne Boland, Pierre Lindenbaum, Thomas Ludwig, Vincent Meyer, Robert Olaso, Lourdes Velo-Suárez, Isabel Alves, Ozvan Bocher, Christian Dina, Anthony F. Herzig, Matilde Karakachoff, Gaëlle Marenne, Aude Saint Pierre, A-L. Leutenegger, H. Perdry

**Affiliations:** 1 Inserm, Univ Brest, EFS, UMR 1078, GGB, 29200 Brest, France; 2 Institute of Genetics and Biophysics A. Buzzati-Traverso - CNR, Naples, Italy; 3 IRCCS Neuromed, Pozzilli, Isernia Italy; 4 LABEX GENMED, Centre National de Recherche en Génomique Humaine, Evry, Paris, France; 5 Inserm, Université Paris Cité, UMR 1141, NeuroDiderot, 75019 Paris, France; 6 CESP Inserm U1018, Université Paris-Saclay, UVSQ, Villejuif, France

**Keywords:** Kinship, Fraternity coefficient, Low-depth, Sequencing data, Genotype likelihoods, Moment estimators

## Abstract

**Background:**

Estimating relatedness is an important step for many genetic study designs. A variety of methods for estimating coefficients of pairwise relatedness from genotype data have been proposed. Both the kinship coefficient $\varphi$ and the fraternity coefficient $\psi$ for all pairs of individuals are of interest. However, when dealing with low-depth sequencing or imputation data, individual level genotypes cannot be confidently called. To ignore such uncertainty is known to result in biased estimates. Accordingly, methods have recently been developed to estimate kinship from uncertain genotypes.

**Results:**

We present new method-of-moment estimators of both the coefficients $\varphi$ and $\psi$ calculated directly from genotype likelihoods. We have simulated low-depth genetic data for a sample of individuals with extensive relatedness by using the complex pedigree of the known genetic isolates of Cilento in South Italy. Through this simulation, we explore the behaviour of our estimators, demonstrate their properties, and show advantages over alternative methods. A demonstration of our method is given for a sample of 150 French individuals with down-sampled sequencing data.

**Conclusions:**

We find that our method can provide accurate relatedness estimates whilst holding advantages over existing methods in terms of robustness, independence from external software, and required computation time. The method presented in this paper is referred to as LowKi (**Low**-depth **Ki**nship) and has been made available in an R package (https://github.com/genostats/LowKi).

**Supplementary Information:**

The online version contains supplementary material available at 10.1186/s12859-022-04795-8.

## Background

Accurate estimates of genetic relatedness between individual organisms are essential for a wide range of study designs and analyses strategies currently at play in plant, animal, or human genetics. These kinship or fraternity coefficients that describe the similarity and extent of shared origin between genomes have a variety of use in genetics. The Genetic Relationship Matrix (GRM) of a sample of individuals is the symmetric matrix of their kinship coefficients. Its eigen-decomposition produces the Principal Components Analysis (PCA) of the sample, which unravels its geographic structure [1]; its use can even be traced back to Cavalli-Sforza who summarised the allelic variations across a few dozen loci by their first principal component [2]. In the Genome Wide Association Studies (GWAS) era, the first principal components have been used to control for population stratification [3]. The GRM is also used as a variance component in Linear Mixed Models, either for controlling population stratification in GWAS [4] or for estimating narrow-sense heritability [5, 6]. Incorporating the matrix of fraternity coefficients in the model allows to compute the dominance component of heritability as well [7, 8]. Furthermore, estimates of the two matrices permit the identification of related individuals in the sample and help characterise the relationships between pairs; for example, the fraternity matrix helps to differentiate sibling pairs from parent–offspring pairs.

These coefficients may currently be estimated in a large variety of ways, and a multitude of methods have been proposed. One’s data characteristics and envisaged analyses will dictate the most appropriate method to be used. For overviews of the current options for relatedness estimation, and its utility, we point the reader to [9–12] and references therein.

In recent years the cost of whole-genome sequencing (WGS) has continued to tumble. Accordingly, more and more study designs have emerged that require large sample sizes to power their analyses. The depth of sequencing carried out over a large sample will have a significant effect on a researcher’s budget. Whilst the accuracy of genotyping is highly dependent on the depth [13], there are often more advantages to being able to sequence a large number of individuals but at a low depth than sequencing far fewer individuals at a high depth. Recent high profile association studies using this approach include [14] and [15]. Low-depth sequencing data were used in many of the cohorts participating in the Haplotype Reference Consortium panel [16] as well. Furthermore, shallow sequencing is often unavoidable in the expanding field of ancient DNA, where the possibilities of sequencing DNA from remains of long deceased organisms [17] are being widely explored. Whilst technological advances allow for greater and greater accuracy in this field, in some circumstances, sequencing to a high depth may simply not be feasible due to the paucity of available genetic material. Another area where genetic material of high quality might be difficult to ascertain is in the study of wild animal populations where DNA is collected from more challenging sources such as hair, feathers, egg membranes or similar [18].

In adaptation to this recent trend of low-depth sequencing studies, a number of methods have been proposed to estimate relatedness coefficients from such datasets. The specificity of these methods is that they work upon genotype likelihoods or posterior probabilities, thus incorporating the uncertainty of genotype calls. These include Hidden Markov Model (HMM) based methods [19], maximum likelihood expectation based methods [20–22], and method-of-moment estimates [23]. The former two approaches can be computationally heavy while moment-based-estimators present a quick and simple alternative. However, the loss of information entailed by analysing genotype likelihoods as a proxy for true genotypes will lead to biased estimates of relatedness which methods using moment-based estimators need to account for.

Moment-based methods have so far only been developed for estimation of the kinship coefficient and SEEKIN, the one software that performs this estimation, requires an intermediate imputation step from an existing HMM method. We propose here LowKi, a method to directly estimate genetic relatedness matrices from genotype likelihoods in a single step, which is available at https://github.com/genostats/LowKi and works in conjunction with the genetic data management and analysis R-package ‘Gaston’ [24].

To assess our approach, we have analysed both simulated and real data. Firstly, we used a simulation dataset which consists of 1,444 individuals with simulated WGS data derived from the complex pedigree structure of the genetic isolates of Cilento [25–27]. This simulation dataset was first produced to assess phasing and imputation methods [28] before being used as a tool to explore heritability estimation [28]. Here we overlay a second layer of data simulation to convert our simulated sequencing data into low-depth sequencing data. To complement our simulation analysis, we also apply our models to a real dataset of 150 individuals from the FranceGenRef [29, 30] WGS panel (LABEX GENMED http://www.genmed.fr/). These individuals have been sequenced to a depth of 30–40$\times$ so we down-sampled individual bam files to create a dataset representative of WGS data at a depth of 2.5$\times$. Finally, we tested our method in other diverse sequencing scenarios using simulated datasets based on haplotypes from the 1000 Genomes Project [31].

Our aim was to show that we can recover relatedness matrices similar to GRMs calculated on high quality genotypes from low-depth data in an expedient manner. We compared our approach to two existing methods which are capable of handling WGS data and accept genotype likelihood data as the input: SEEKIN (v1.01) [23] and NGSRelateV2 (v2) [20, 21]. LowKi calculates moment estimates of kinship and fraternity in the form of a genetic relatedness matrices (GRMs) with suitable adjustments for the genotype uncertainty that is present with low-depth WGS data. SEEKIN provides moment-estimators for kinship only, using a similar approach to LowKi but requiring an intermediate imputation step, typically performed by the software BEAGLE [32]. NGSRelateV2 uses maximum likelihood estimation and computes a wider range of relatedness statistics (kinship, fraternity, inbreeding, and Jacquard’s 9 identity coefficients). It can typically be used in conjunction with ANGSD [33], a bioinformatics suite for handling both raw and previously-processed sequencing data. We show here that LowKi’s estimates are competitive in terms of accuracy and less time consuming to compute than those provided by alternative software.

## Results

### Overview of LowKi

For a pair of individuals $i$ and ${i}^{^{\prime}}$, LowKi provides estimates of ${\varphi }^{i{i}^{^{\prime}}}$, the kinship coefficient of individuals $i$ and ${i}^{^{\prime}}$. This is the probability that a pair of randomly drawn alleles from individual $i$ and ${i}^{^{\prime}}$ at the same locus will be in a state of Identity-by-Descent (IBD). LowKi also provides a moment-estimate of ${\psi }^{i{i}^{^{\prime}}}$ the fraternity coefficient which is the proportion of the genome for which individuals $i$ and ${i}^{^{\prime}}$ share two pairs of alleles at the same locus (IBD = 2).

In the development of LowKi, first ‘naïve’ moment estimators were defined by an approximation of the construction of the classical moment estimators used for genotype data; but based on individual genotype likelihoods. These estimators, which are referred to as ‘unadjusted estimates’ in this study, were observed to be biased. We observed that as the average read depth decreases, the observed bias increases. It is indeed intuitive that additional uncertainty or ‘fuzziness’ in the genotype likelihoods gives a stronger downward bias in a moment-estimate. This makes sense when considering that the additional fuzziness represents an increasing lack of information about the genotypes as random error contributions to the genotype likelihoods (occurring independently between individuals) become more and more prevalent. We fit regression models between point-wise moment estimators and a summary statistic of the genotype likelihood fuzziness to obtain LowKi’s final estimates (denominated in the text as ‘adjusted estimates’). A full description of the LowKi calculation and bias correction are presented in the Methods.

### Relatedness estimation from genotype likelihoods in CilentoSim

Our primary simulation dataset (here denoted as ‘CilentoSim’ and described in the Methods) comprises 1444 individuals and 490,995 genetic variants across the 22 autosomal chromosomes. We established that this variant set was appropriate for the calculation of a GRM as this set captured the known pedigree structure of Cilento. This is seen by comparing the kinship and fraternity GRMs (calculated from the simulated genotypes) to the true IBD sharing matrices calculated based on records of all haplotype mosaics created in the simulation (see 'Methods') (Additional file 1: Fig. S1). For kinship, the genotype-based GRM gave a very precise estimate of the exact simulated IBD-sharing fractions. For fraternity, the GRM estimates are highly correlated with the simulated IBD-sharing but we observed lower precision compared to kinship.

We artificially reduced the depth of our simulated sequencing data by drawing random alleles from each simulated individual genotype to a specified depth and then replacing, in our simulation, each true genotype with three genotype likelihoods. The method used here is based on the simulation proposed by Kim et al. [34], uses a simplified version of the genotype likelihood model of GATK [35–37], and is described fully in the Methods. We used this additional layer of simulation to give new datasets with average read depths of 2.5$\times$, 5$\times$, and 10$\times$.

We applied our method LowKi alongside SEEKIN and NGSRelateV2. We chose to compare the two moment estimators, LowKi and SEEKIN to the ‘Full GRM’ estimates obtained from complete simulated genotypes, which is the best estimation a moment estimate can achieve. It was more meaningful to compare NGSRelateV2, which is a maximum likelihood estimator, to the simulated IBD sharing probabilities (which are similar but not identical to the Full GRM estimates, see Additional file 1: Fig. S1a).

**Fig. 1:**
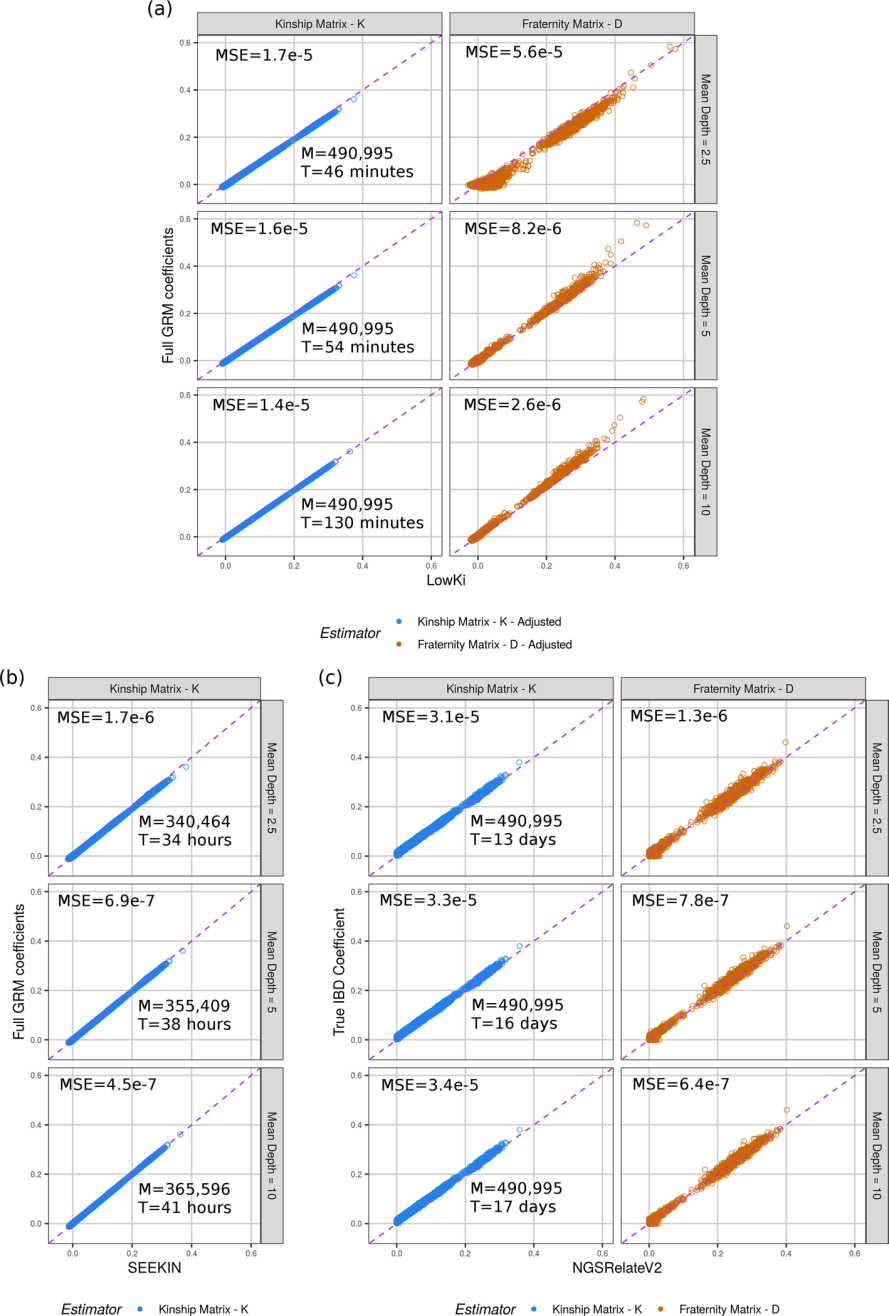
**a** LowKi estimates for kinship and fraternity for CilentoSim. Off-diagonal elements of the estimated kinship and fraternity matrices against the true simulated IBD sharing coefficient in CilentoSim at three different simulated mean read depths (2.5$\times$, 5$\times$, and 10$\times$). The number of variants (M) and the time (T) required for the calculation of both kinship and fraternity matrices are overlaid on the figure (for SEEKIN the time refers only to the calculation of kinship). The mean-squared error (MSE) between the estimators and their respective benchmarks are also given. **b–c** Corresponding estimates from SEEKIN (kinship only) and NGSRelateV2

In Fig. 1a and b the off-diagonal coefficients of the relationship matrices estimated by LowKi and SEEKIN, respectively, are thus compared against the ‘Full GRM’ estimates, while in Fig. 1c the coefficients computed by NGSRelateV2 are compared to the IBD sharing probabilities. This choice of different reference coefficients has virtually no effect in the case of kinship coefficients, but when it comes to fraternity coefficients, there’s some sizeable differences between Full GRM estimates and IBD sharing probabilities; for this reason, we also compared LowKi estimates to the later, see Additional file 1: Fig. S1b.

LowKi is able to recover the structure of the Full GRM kinship and fraternity matrices (Fig. 1a); SEEKIN and NGSRelateV2 also performed strongly. The mean-squared error (MSE) between the estimators and their respective benchmarks are also given in Fig. 1a–c showing that all methods increased their precision as the simulated mean depth increased. Run-times and number of SNPs considered are also given. In Additional file 1: Fig. S2, Fig. 1a is repeated with the inclusion of the ‘Unadjusted Estimates’ from our model that are downwardly biased by a multiplicative factor; demonstrating the efficiency and necessity of the adjustment procedure and the previously described observation that the magnitude of the bias increased as the average read depth decreased. While the bias adjustment procedure performs well, the discrepancy between the Full GRM fraternity coefficients and the true IBD sharing, visible on the first row of Additional file 1: Fig S1a, hampers the performance of LowKi for these coefficients, as seen in Additional file 1: Fig S1b. This phenomenon might be exacerbated in this particular dataset, in which there is a substantial level of inbreeding, which is not accounted for by method-of-moment estimates.

Finally, it was observed that at 10$\times$ LowKi slightly underestimated the fraternity coefficients in CilentoSim. Further simulations were performed (see Additional file 1: Fig. S3a–b) to investigate this observation. It was seen that whilst there was no issue for estimating kinship, fraternity would be underestimated by LowKi when analysing genotype-likelihoods from relatively high-depth data (10$\times$ and beyond). However, above 10$\times$, GRMs based on hard-called genotypes perform well for estimating fraternity and so there would not be an advantage in using LowKi in any case.

### Comparison to existing software on simulated data

SEEKIN only produces an estimate for the kinship matrix and indeed uses in part a similar moment-estimator to our method presented here. SEEKIN gave very accurate kinship estimates. The key specificities of SEEKIN involve an intermediate step of the imputation software BEAGLE (v4.1) [32], the leveraging of an external reference panel (here the 1000 Genomes Project phase 3 haplotype reference panel [31] was used) and a re-weighting based on the imputation quality of variants in the summation that forms each GRM entry. As the initial step of BEAGLE cannot be avoided, we included the runtime of BEAGLE into the runtime of SEEKIN. For low-depth data, running BEAGLE is very time consuming. We followed the recommendations for using BEAGLE as described by the authors of SEEKIN. The use of BEAGLE will change the data in two particular ways: firstly, the uncertainty present in the initial data will be largely removed as BEAGLE will effectively take the prior information given to it in the form of genotype likelihoods and add precision based on similarities between pairs of individuals in the sample, or between individuals in the sample and the external panel of reference haplotypes, using the same haplotype clustering HMM machinery as is applied in BEAGLE’s haplotype phasing and genotype imputation methods. Secondly, running BEAGLE is likely to imply the removal of some variants. For example, on our simulated dataset with genotype likelihoods created using a mean depth of 2.5$\times$, for our initial dataset of 490,995, BEAGLE returns information for only 340,464 variants. In Additional file 1: Fig. S4, the difference in precision is displayed between the initial genotype likelihoods supplied to BEAGLE (for a random selection of 25,000 variants) and the posterior genotype probabilities. This demonstrates the importance of the use of BEAGLE to the SEEKIN method.

A different and more refined approach is proposed by NGSRelateV2 which directly estimates relatedness parameters through maximum likelihood estimation. Indeed, for the fraternity matrix, the true IBD coefficients were estimated more precisely with NGSRelateV2 than with a GRM on simulated genotypes (Additional file 1: Fig. S1). This software also produces additional information as it gives estimates for all nine condensed identity-by-descent states. NGSRelateV2 gave very accurate estimates for both kinship and fraternity in the CilentoSim analysis though did require extensive amounts of running time on default settings. NGSRelateV2 is multithreaded and uses four threads as a default; we did not alter this default setting. Using more than the four default threads would give an increase in speed but this software will remain computationally expensive for large sample sizes.

### Testing with real data

We also applied our method, SEEKIN, and NGSRelateV2 to a set of real genotypes. 150 individuals with WGS data were made available to us from the FranceGenRef panel; of which all individuals are not closely-related except for two pairs of siblings. We down-sampled this dataset from 30–40$\times$ to 2.5$\times$ in order to create realistic low-depth WGS data. The estimates of the Kinship and Fraternity matrix entries for these two sibling pairs are given in Table 1. We also include the unadjusted estimates of LowKi to demonstrate the initial bias affecting its moment estimates that the adjustment procedure aims to correct. Moment-estimators on down-sampled data are to be compared against moment-estimators on the original 30–40$\times$ data, while estimates from NGSRelateV2 on down-sampled data are more pertinently compared to the estimates of NGSRelateV2 applied the original 30–40$\times$ data. Having observed in the simulation study that GRM estimates of fraternity based on genotypes from WGS data (Full GRM) may not necessarily agree with NGSRelateV2 (which in fact gave superior estimates—Additional file 1: Fig. S1), it would not be meaningful to benchmark NGSRelateV2 on low-depth data against Full GRM estimates. Furthermore, GRM estimates rely on naively estimating minor-allele frequency from within the sample. As 150 is a relatively small sample size, this was an additional reason to expect a different baseline in estimates between a Full GRM and NGSRelateV2 (which has a specific internal mechanism for estimating minor-allele frequencies). This was indeed the case as seen in the analysis of WGS data at 30–40$\times$ in Table 1.

**Table 1:** Analysis of FranceGenRef data. $\widehat{\mathrm{\varphi }}$ is the estimate of the two siblings’ unobserved kinship coefficient $\mathrm{\varphi }$

		WGS data at 30–40×		Down-sampled WGS data at ~ 2.5×				
		Gaston Full GRM	NGS- RelateV2	LowKi (Unadjusted)*	LowKi	BEAGLE + LowKi	BEAGLE + SEEKIN	NGS- RelateV2‡
Sibling Pair 1	$\widehat{\varphi }$	0.258	0.268	0.122	0.278	0.265	0.268	0.256
	$\widehat{\psi }$	0.294	0.379	0.059	0.185	0.255	†	0.459
Sibling Pair 2	$\widehat{\varphi }$	0.216	0.228	0.100	0.223	0.225	0.226	0.216
	$\widehat{\psi }$	0.196	0.270	0.035	0.115	0.166	†	0.367
M (number of variants)				1,009,181	1,009,181	949,075	949,075	1,009,181
Time				4 min	4 min	15 h**	15 h **	6 h

$\widehat{\psi }$ is the estimate of the two siblings’ unobserved fraternity coefficient $\psi$. (*) Unadjusted values from LowKi, similar to in Additional file 1: Fig. S2. (**) The time required for these estimators is almost entirely due to BEAGLE, both LowKi and SEEKIN require only a few minutes. (†) SEEKIN does not provide estimates of $\psi$. (‡) NGSRelateV2 results on down-sampled data should be compared to results from the same software but applied to the original data that had not been down-sampled

All methods were able to distinguish the two sibling pairs as being closely related compared to all other pairs (Additional file 1: Fig. S5). All methods produced accurate estimates for the kinship coefficient but the fraternity coefficient proved difficult for both LowKi and NGSRelateV2 to estimate accurately. One intuitive solution is to use the same approach as SEEKIN and first perform imputation with BEAGLE; this allowed us to improve our estimates of the fraternity coefficients between the two sibling pairs (Additional file 1: Fig. S5, panel (c)) at the cost of incurring a long run time equivalent to the BEAGLE + SEEKIN strategy. In every case, LowKi underestimates both fraternity coefficients; however, NGSRelateV2 appeared to give overestimations compared to its estimates on WGS data at 30–40× for the same sibling pairs (Table 1, Additional file 1: Fig. S5).

### Testing other sequencing scenarios

We created further simulated datasets using the data from the 1000 Genomes Projects to test LowKi in additional and more diverse settings. We wished to assess the performance of LowKi in the case of very low depth data (Additional file 1: Figs. S6–7) and in the case of a sample containing individuals sequenced at different sequencing depths (Additional file 1: Fig. S8). In both of these analyses, we also applied NGSRelateV2. Furthermore, we also tested LowKi’s performance for a very small sample size of just 20 individuals (Additional file 1: Fig. S9).

To test data of very low depth, we simulated 200 individuals including a small proportion of related pairs, and 200,000 SNPs with a mean read-depth that varied between 0.1× and 3× . Details of the simulation set-up are given in the Methods. As in previous analyses, LowKi is benchmarked against Full GRM estimates, and NGSRelateV2 is benchmarked against simulated IBD-sharing (Additional file 1: Figs. S6–7). We observed that LowKi was able to give a reasonable estimate of the kinship at all depths tested. For fraternity however, below 1× it was not possible to distinguish unrelated and related pairs. NGSRelateV2 also performed well for kinship at all depths but equally struggled to correctly estimate fraternity at very low-depths on the same data. Indeed, for fraternity, LowKi gave under-estimations whereas NGSRelateV2 tended to give over-estimations which corresponds with our analysis of the two sibling pairs in the real data example FranceGenRef (Table 1).

To simulate a sample with variable read-depths per individual, we again simulated 200 individuals and 200,000 SNPs, and specified four groups of 50 individuals that would have mean-depths of 2×, 3×, 4×, and 5× respectively. The model of LowKi does not take into account such within-sample heterogeneity; nevertheless, reasonable estimations of kinship and fraternity were recovered (Additional file 1: Fig. S8) though the precision was lower than when analysing a sample with uniform read-depths. NGSRelateV2 also coped well with this mixture of different read-depths but also gave much less precise estimations compared to other analyses (eg. Fig. 1c) where comparable read-depths were used. We would advise caution in applying LowKi to datasets containing individuals with very different sequencing depths as this heterogeneity is not taken into account. One potential solution for this scenario could be to implement imputation using BEAGLE in order to smooth-out the heterogeneity between different samples; though of course this would imply a much greater computational time.

To test LowKi for small sample sizes, we created a sample of 20 individuals with a simulated depth of 2×. Running LowKi on these 20 individuals alone gave imprecise estimates but an improvement could be attained by harnessing minor-allele frequencies estimated from a 2nd independent group of 100 individuals simulated in parallel under the same settings (Additional file 1: Fig. S9). NGSRelateV2 was also tested on this small sample and also provided with the same external minor allele frequencies; as with LowKi, imprecise results were attainted. In the case of small sample sizes, wherein allele frequencies cannot be accurately estimated, we would advise that this external allele-frequency option be used. This would necessitate first obtaining appropriate allele frequencies from an external source, but would give better results than the default settings of LowKi which were conceived to provide fast kinship and fraternity estimates from large sample sizes. Note that LowKi allows to calculate maximum likelihood estimates of minor allele-frequencies [34] from external data (low-depth or otherwise) to facilitate the analysis of small sample sizes.

## Discussion

It is intuitive that with data of the huge breadth of the whole human genome, even when the quality of sequencing data is extremely low, relatedness between individuals should still be captured. Existing methods have either involved maximum likelihood estimation or moment-estimators of relatedness coefficients. The former estimators carry a high computation burden and require a modelling of the mechanism that links true genotypes to genotype likelihoods. The latter, moment estimators, have a lower computational burden, but will however suffer from bias. This can either be dealt with using an intermediate imputation algorithm to improve the data as is the case of SEEKIN that requires BEAGLE; or by attempting to explicitly account for the bias as in the method we have developed here. LowKi represents very rapid moment-estimates of kinship and fraternity from large samples of individuals sequenced at low-depth, whilst remaining competitive in terms of accuracy with leading software NGSRelateV2 which uses more complex maximum likelihood methods. It is worth noting however that NGSRelateV2’s accuracy is better than LowKi’s, which reflects the general superiority of maximum likelihood estimates over method-of-moments estimates.

By estimating orthogonal components for additive and non-additive genotypic effects, we constructed a moment estimator for the kinship and the fraternity coefficient from low-depth data. Such a moment-estimator has never been provided for fraternity by an alternative software. Estimation of fraternity is important for classifying relatives and also for exploring the effects of non-additive genetic effects [8]. Our moment-estimators for fraternity were sufficient to distinguish pairs of siblings in the analysis of FranceGenRef where even NGSRelateV2 returned slightly imprecise estimates of fraternity from data down-sampled to 2.5$\times$. For the fraternity matrix our re-adjusted estimators were not as accurate as for kinship, though it was clear that fraternity coefficients are harder to estimate and are not always perfectly estimated with moment-estimators even when using genotype data. Both LowKi and NGSRelateV2 struggled to estimate fraternity from data with very low sequencing depths. Globally, NGSRelateV2 performed very strongly in our study, particularly in the large and complex scenario of the large simulated isolated population dataset based on the Cilento isolates. The key drawback was that longer computational times were required for NGSRelateV2; whereas LowKi’s moment estimators could be attained relatively quickly.

To correct for bias in the estimates of LowKi, we introduced an innovative yet simple regression-based approach. The adjustment method proposed should be robust to different sources of bias arising from genotype uncertainty coming from different types of bioinformatics pipeline. This could give more flexibility than likelihood-based methods such as lcMLkin [22] or NGSRelateV2, as well as similar methods proposed for estimating inbreeding coefficients [19]. Note that we did not test lcMLkin here following its assessment in the publication presenting SEEKIN [23]. The adjustment technique developed here for LowKi could be harnessed in other areas of research involving low-depth sequencing data.

We showed that the alternative to such bias-correction, using an external imputation algorithm, could also lead to lengthy run times and a reliance on the accuracy of the external algorithm. Notwithstanding such observations, the methodology of SEEKIN that uses the intermediate step of BEAGLE is clearly highly effective and could also be used in conjunction with LowKi. Indeed, BEAGLE probably worked particularly well in the case of our CilentoSim dataset due to the many pairs of very closely related individuals in the sample. However, in the circumstance where only a small sample size is present or when an appropriate reference panel cannot be ascertained (both might be the case in studies of ancient DNA or small isolated populations for examples), it is beneficial to have a method for proceeding directly to relatedness estimates from genotype likelihood data; which LowKi provides.

## Conclusion

LowKi was effective at computing accurate kinship and fraternity matrices in a large sample of individuals with a full spectrum of IBD-sharing between pairs of related individuals in a detailed simulation study. We complemented this analysis by assessing an example of real low-depth genetic data from FranceGenRef, where our re-adjusted relatedness coefficient estimates were able to quickly and accurately identify the pairs of siblings in the sample. By analysing real data, we have illustrated that our estimators perform well outside of the idealised setting of a simulation. Real data will harbour phenomena such as allele-balance bias [38] or region-specific sequencing error rates [39] so it was important to verify our estimators on an example of true sequencing data.

When compared to existing methods, LowKi does not require the use of intermediate software such as BEAGLE and thus requires by far the least computation time. The innovative adjustment method applied in LowKi gives flexibility to the method to account for different possible sources of bias. The LowKi methods proposed here for estimating relatedness have been made available at https://github.com/genostats/LowKi and work in conjunction with the existing R-package Gaston. This represents a fast and accurate standalone option for computing kinship and fraternity coefficients from low-depth sequencing data.

## Methods

Throughout, the index $i\in 1,\dots ,N$ will denote individuals (with two different individuals denoted as $i$ and ${i}^{^{\prime}}$) and $j\in 1,\dots ,M$ will indicate bi-allelic genetic variants. Individual level genotype data are denoted as ${G}^{ij}$ which take values in $\left\{\mathrm{0,1},2\right\}$ for the three possible genotypes $AA,Aa$, and $aa$, respectively.

### Simulation of low-depth data

Existing simulated WGS data for 1,444 individuals based on the pedigree of the Cilento isolates was our starting point [8]. These simulated individuals were constructed as mosaics of haplotype chunks sourced from the UK10K imputation panel. The formation of mosaic haplotypes from the UK10K imputation reference panel [40] has been described in two previous studies [8, 28]. The individuals share chunks in accordance with the known pedigree of Cilento by means of gene-dropping [41] onto the pedigree. By recording the source of each chunk (within the UK10K), we have knowledge of the exact IBD-sharing probabilities in the simulated population. For this study, we added an additional layer of simulation to translate simulated genotypes into simulated genotype-likelihoods typical of low-depth WGS data. We also only retained 490,995 variants by first selecting those with a minor allele frequency above 5% and then by performing pruning on linkage disequilibrium with Gaston.

In the Cilento cohort, there are 19 individuals with WGS data. These individuals were sequenced to an average depth of 50–60$\times$. From this dataset, we took a list of per-variant mean read depths and scaled each entry so that the global mean read depth would either be 10, 5, or 2.5. These lists became the lists of mean depths for each variant for our simulation. For each individual level genotype ${G}^{ij}$ and for an assigned average read depth ${d}_{j}$ for the position, we draw three sets of reads to represent the number of reads carrying the reference allele $A$, the minor allele $a$, and error reads that carry a base that matches neither $A$ or $a.$ The size of these three groups are denoted as ${R}_{A}$, ${R}_{a},$ and ${R}_{\varepsilon }$. We model the occurrence of reads with Poisson distributions and thus draw ${R}_{A}$ as Poisson with parameter ${\rho }_{A}^{ij}{d}_{j}$, ${R}_{a}$ as Poisson with parameter ${\rho }_{a}^{ij}{d}_{j},$ and ${R}_{\varepsilon }$ as Poisson with parameter ${\rho }_{\varepsilon }^{ij}{d}_{j}$. These parameters have values depending on the true genotypes ${G}^{ij}$ and the error rate ${\varepsilon }_{j}$ at the position as shown in Table 2.

**Table 2:** Parameters of the Poisson distribution for reads carrying alleles A, a, and error reads, depending on the true genotypes ${\mathrm{G}}^{\mathrm{ij}}$ and the error rate ${\upvarepsilon }_{\mathrm{j}}$

	${G}^{ij}=0$ Genotype AA	${G}^{ij}=1$ Genotype Aa	${G}^{ij}=2$ Genotype aa
${\rho }_{A}^{ij}$	$1-{\varepsilon }_{j}$	$\frac{1}{2}\left(1-{\varepsilon }_{j}\right)$+${\frac{1}{6}\varepsilon }_{j}$	${\frac{1}{3}\varepsilon }_{j}$
${\rho }_{a}^{ij}$	${\frac{1}{3}\varepsilon }_{j}$	$\frac{1}{2}\left(1-{\varepsilon }_{j}\right)$+${\frac{1}{6}\varepsilon }_{j}$	$1-{\varepsilon }_{j}$
${\rho }_{\varepsilon }^{ij}$	${\frac{2}{3}\varepsilon }_{j}$	${\frac{2}{3}\varepsilon }_{j}$	${\frac{2}{3}\varepsilon }_{j}$

The values of ${\varepsilon }_{j}$ were drawn randomly as ${10}^{-{u}_{j}}$ with ${u}_{j}$ drawn uniformly between 2 and 3. In any case where ${R}_{A}={R}_{a}=0$, we set the all three genotype likelihoods as a missing genotype. In order to compute genotype likelihoods, we apply a flat prior and binomial likelihoods as used in the simplest interpretation of the GATK calling algorithm. This leads to the likelihood of the observed reads occurring given the true genotypes as proportional to ${\left({\rho }_{A}^{ij}\right)}^{{R}_{A}}\times {\left({\rho }_{a}^{ij}\right)}^{{R}_{a}}{\times \left({\rho }_{\varepsilon }^{ij}\right)}^{{R}_{\varepsilon }}$.

### Moment estimators of relatedness from low-depth

In order to define our new moment-estimators for relatedness matrices, we give first a brief introduction and explanation of notations and theory. Here, the concepts of additive and non-additive components are being borrowed from the literature of quantitative genetics and in particular the polygenic models first proposed by RA Fisher [42] where the genetic effects of each variant can be split into two orthogonal components. The first being the additive contribution, describing the effect that increases linearly with the number of minor alleles in the genotype, and the second being the non-additive contribution which describe the deviations away from the additive model caused by interactions between the two alleles and a single locus as is observed for example in recessive or dominant models.

Genotype-based GRM estimates for kinship and fraternity matrices (denoted as $K$ and $D$, respectively) can be defined as follows:

$${K}_{i{i}^{^{\prime}}}=\frac{1}{M}{\sum }_{j=1}^{M}{X}_{A}^{ij}\times {X}_{A}^{{i}^{^{\prime}}j} \quad and \quad {D}_{i{i}^{^{\prime}}}=\frac{1}{M}{\sum }_{j=1}^{M}{X}_{D}^{ij}\times {X}_{D}^{{i}^{^{\prime}}j}$$

where ${X}_{A}^{ij}$ and ${X}_{D}^{ij}$ are the classical additive and non-additive components of the individual level genotypes ${G}^{ij}$ which are defined as follows:

$${X}_{A}^{ij}={\alpha }_{0}^{j} {1}_{\left\{{G}^{ij}=0\right\}}+{\alpha }_{1}^{j} {1}_{\left\{{G}^{ij}=1\right\}}+{\alpha }_{2}^{j} {1}_{\left\{{G}^{ij}=2\right\}}$$

$${X}_{D}^{ij}={\delta }_{0}^{j} {1}_{\left\{{G}^{ij}=0\right\}}+ {\delta }_{1}^{j} {1}_{\left\{{G}^{ij}=1\right\}}+{\delta }_{2}^{j} {1}_{\left\{{G}^{ij}=2\right\}}$$

where

1 $${\alpha }_{k}^{j}=\frac{k-2{q}_{j}}{\sqrt{2{p}_{j}{q}_{j}}}, \left(k=0, 1, 2\right)\quad and\quad {\delta }_{0}^{j}=\frac{{q}_{j}}{{p}_{j}}, {\delta }_{1}^{j}= -1, {\delta }_{2}^{j}=\frac{{p}_{j}}{{q}_{j}},$$

${q}_{j}$ being the minor allele frequency of variant $j$ and${p}_{j}=1-{q}_{j}$. Alternative notations are presented in [7] and [43] but give the same moment-estimators. The values of $({\alpha }_{0}^{j},{\alpha }_{1}^{j},{\alpha }_{2}^{j})$ are obtained through standardisation of${G}^{\cdot j}$, interpreted as a random variable (the SNP index $j$ is fixed, the sample is constituted of the values ${G}^{ij}$ for$i=1,\dots , N$): its expected value is $2{q}_{j}$ and its standard deviation is $\sqrt{2{p}_{j}{q}_{j}}$ (assuming Hardy–Weinberg proportions). The resulting random variable ${X}_{A}^{\cdot j}$ has expected value 0 and variance 1. The values of $({\delta }_{0}^{j},{\delta }_{1}^{j},{\delta }_{2}^{j})$ can then be determined by imposing three constraints on the resulting variable ${X}_{D}^{\cdot j}$:$E\left({X}_{D}^{\cdot j}\right)=0$,$var({X}_{D}^{\cdot j})=1$, and $E\left({X}_{A}^{\cdot j} {X}_{D}^{\cdot j}\right)=0$ (the two variables are independent—or ‘orthogonal’—in the sample).

It is well established that under the circumstances of correct Hardy–Weinberg proportions in the population and of having in-hand the correct value of the minor allele frequencies, ${K}_{i{i}^{^{\prime}}}$ will be an unbiased estimator of $2{\varphi }^{i{i}^{^{\prime}}}$ and ${D}_{i{i}^{^{\prime}}}$ will be an unbiased estimator of ${\psi }^{i{i}^{^{\prime}}}$. Such genetic relatedness matrices were first introduced in [44, 45] for kinship and in [5] for heritability and have been repurposed for many other uses.

Such moment estimators necessitate allele frequency information. For low-depth sequencing data, it is possible to estimate allele frequencies directly from genotype probabilities. This is however problematic as the additional uncertainty in the data will characteristically lead to increased estimates of allele frequencies as well as potential perturbations to Hardy–Weinberg proportions. This can be observed in Additional file 1: Fig. S10 where we compared observed minor alleles frequencies and heterozygosity statistics from the original simulated genotypes of CilentoSim against those estimated from genotype likelihoods at a depth of 2.5×. The perturbation to allele frequencies is difficult to avoid, but the issue of potential Hardy–Weinberg deviations may be circumvented by defining our additive and non-additive components on estimated genotype frequencies (rather than allele) in order to correctly achieve orthogonality. The derivations that we give here are equivalent to those found in Vitezica et al. [43]. Not assuming Hardy–Weinberg equilibrium may also aid LowKi to be robust to inbreeding in the sample, but as a moment estimator, LowKi does not specifically account for inbreeding whereas NGSRelateV2 does.

Across the sample, we estimate genotype probabilities by averaging across all genotype probabilities in the sample. First, individual genotype likelihood data (typically available on a log-scale) in the form${GL}_{AA}^{ij}$, ${GL}_{Aa}^{ij},$ and ${GL}_{aa}^{ij}$ are rescaled to genotype probabilities${P}_{AA}^{ij}$, ${P}_{Aa}^{ij},$ and${P}_{aa}^{ij}$. Then we estimate genotype frequencies in the sample as: ${\overline{P}}_{AA}^{j}=\frac{1}{N}{\sum }_{i=1}^{N}{P}_{AA}^{ij}$, ${\overline{P}}_{Aa}^{j}=\frac{1}{N}{\sum }_{i=1}^{N}{P}_{Aa}^{ij}$, and${\overline{P}}_{aa}^{j}=\frac{1}{N}{\sum }_{i=1}^{N}{P}_{aa}^{ij}$.

The additive and dominant component are defined as:

$${\tilde{X }}_{A}^{ij}={\stackrel{\sim }{\alpha }}_{0}^{j} {P}_{AA}^{ij}+ {\stackrel{\sim }{\alpha }}_{1}^{j} {P}_{Aa}^{ij}+ {\stackrel{\sim }{\alpha }}_{2}^{j} {P}_{aa}^{ij}$$

$${\tilde{X }}_{D}^{ij}={\stackrel{\sim }{\delta }}_{0}^{j} {P}_{AA}^{ij}+ {\stackrel{\sim }{\delta }}_{1}^{j} {P}_{Aa}^{ij}+ {\stackrel{\sim }{\delta }}_{2}^{j} {P}_{aa}^{ij}$$

As previously, the values of the triplet $({\stackrel{\sim }{\alpha }}_{0}^{j}, {\stackrel{\sim }{\alpha }}_{1}^{j}, {\stackrel{\sim }{\alpha }}_{2}^{j})$ are obtained by standardizing the vector with (0, 1, 2) using the observed mean and variance of the expected minor allele count (or genotype dosage) ${\tilde{G }}^{\cdot j}$ which is constituted of the values ${\tilde{G }}^{ij}$ for $i=1,\dots , N$, where ${\tilde{G }}^{ij}$ = ${P}_{Aa}^{ij}+2{P}_{aa}^{ij}$. The values of $({\stackrel{\sim }{\delta }}_{0}^{j}, {\stackrel{\sim }{\delta }}_{1}^{j}, {\stackrel{\sim }{\delta }}_{2}^{j})$ are derived from the constraints $E\left({\tilde{X }}_{D}^{\cdot j}\right)=0$, $var({\tilde{X }}_{D}^{\cdot j})=1$, and $E\left({\tilde{X }}_{A}^{\cdot j} {\tilde{X }}_{D}^{\cdot j}\right)=0$, where, as before, expected values are computed across the sample ($j$ is fixed and $i$ goes from $1$ to $N$). We obtain

$$\left({\stackrel{\sim }{\alpha }}_{0}^{j}, {\stackrel{\sim }{\alpha }}_{1}^{j}, {\stackrel{\sim }{\alpha }}_{2}^{j}\right)={\left({\overline{P}}_{Aa}^{j}+4{\overline{P}}_{aa}^{j}{\overline{P}}_{AA}^{j}-{{\overline{P}}_{Aa}^{j}}^{2}\right)}^{-\frac{1}{2}}\times \left(-{\overline{P}}_{Aa}^{j}-2{\overline{P}}_{aa}^{j}, 1-{\overline{P}}_{Aa}^{j}-2{\overline{P}}_{aa}^{j}, 2-{\overline{P}}_{Aa}^{j}-2{\overline{P}}_{aa}^{j}\right)$$

and

$$\left({\stackrel{\sim }{\delta }}_{0}^{j}, {\stackrel{\sim }{\delta }}_{1}^{j}, {\stackrel{\sim }{\delta }}_{2}^{j}\right)={\left({\overline{P}}_{aa}^{j}+4\frac{{\overline{P}}_{aa}^{j}{\overline{P}}_{AA}^{j}}{{\overline{P}}_{Aa}^{j}}+{\overline{P}}_{AA}^{j}\right)}^{-\frac{1}{2}}\times \left(\sqrt{\frac{{\overline{P}}_{aa}^{j}}{{\overline{P}}_{AA}^{j}}} , -2\sqrt{\frac{{\overline{P}}_{aa}^{j}{\overline{P}}_{AA}^{j}}{{{\overline{P}}_{Aa}^{j}}^{2}}} ,\sqrt{\frac{{\overline{P}}_{AA}^{j}}{{\overline{P}}_{aa}^{j}}}\right).$$

Finally, the GRM matrices using genotype likelihoods are computed as

$${\tilde{K }}_{i{i}^{^{\prime}}}=\frac{1}{M}{\sum }_{j=1}^{M}{\tilde{X }}_{A}^{ij}\times {\tilde{X }}_{A}^{{i}^{^{\prime}}j}\quad and\quad{\tilde{D }}_{i{i}^{^{\prime}}}=\frac{1}{M}{\sum }_{j=1}^{M}{\tilde{X }}_{D}^{ij}\times {\tilde{X }}_{D}^{{i}^{^{\prime}}j}.$$

### Implementation

These estimators for kinship and fraternity have been implemented in the R-package LowKi; for which the majority of the code is written in C++ to provide fast computation times. A vignette for testing LowKi has been made available, where toy datasets are provided which were created from haplotypes of the 1000 Genomes Project and the haplotype mosaic simulator Mozza which allows for the simulation of low-depth WGS data.

Presented here are the default moment estimators of LowKi. However, LowKi also accepts user specified estimations of minor-allele frequencies with a slight change in the moment estimator calculations: In the case where external allele-frequencies are used, Hardy–Weinberg equilibrium is assumed and the components $\left({\stackrel{\sim }{\alpha }}_{0}^{j}, {\stackrel{\sim }{\alpha }}_{1}^{j}, {\stackrel{\sim }{\alpha }}_{2}^{j}\right)$ and $\left({\stackrel{\sim }{\delta }}_{0}^{j}, {\stackrel{\sim }{\delta }}_{1}^{j}, {\stackrel{\sim }{\delta }}_{2}^{j}\right)$ reduce and become equivalent to the ‘classical’ ones given in Eq. 1.

Furthermore, we have equipped LowKi with the functionality to calculate maximum-likelihood estimates of such frequencies from external datasets (or potentially from within the study sample) using a simplified version of the method presented in Kim et al. [34].

### Correcting the bias

Our initial simulation results indicated a clear relationship between the average depth and the biases in the estimates of both off-diagonal and diagonal elements in the GRMs. Indeed, the bias observed appeared similar to the bias that occurs when hard-called genotypes (setting the genotypes to the most probable genotype) are used for estimating GRMs as reported by Dou et al. [23]. For a given average read depth, our simulation results suggest that $E\left[{\tilde{K }}_{i{i}^{^{\prime}}}\right]=2{\beta }_{1}{\varphi }_{i{i}^{^{\prime}}}$ and $E\left[{\tilde{D }}_{i{i}^{^{\prime}}}\right]={\beta }_{2}{\psi }_{i{i}^{^{\prime}}}$ for some unknown constants ${\beta }_{1}$ and ${\beta }_{2}$.

Each off-diagonal element of matrices $\tilde{K }$ and $\tilde{D }$ is itself an average over many point estimates from individual genetic variants. These point estimates come from genetic variants with differing read depths and qualities and hence we should expect some variants to be giving greater or lesser biased point-wise estimates. When the depth is low, the three genotype probabilities tend to become less certain, we move further away from a tuple of probabilities such as $\left(\mathrm{1,0},0\right)$ (which represents a certain genotype of $AA$) towards a tuple such as $\left(\frac{1}{2},\frac{1}{2},0\right)$ or even $\left(\frac{1}{3},\frac{1}{3},\frac{1}{3}\right)$ where there is no certainty as to what the true genotype may be. This uncertainty or ‘fuzziness’ of the data can be summarised by the variance of the genotype (here thought of as a random variable taking values in {0,1,2} occurring at probabilities ${P}_{AA}^{ij}$, ${P}_{Aa}^{ij},$ and ${P}_{aa}^{ij}$, respectively. We denote this measure as ${\upsilon }^{ij}{:=}$ ${P}_{Aa}^{ij}\left(1-{P}_{Aa}^{ij}\right)+4{P}_{aa}^{ij}\left(1-{P}_{aa}^{ij}\right)-4{P}_{Aa}^{ij}{P}_{aa}^{ij}$. To demonstrate the relationship between this fuzziness and the bias in relatedness estimates, we repeatedly simulated low-depth data for a single variant shared (with IBD status at random) by two siblings; varying values of the average depth and minor allele frequency for the variant. Pairs of siblings are expected to share at least one haplotype IBD for 50% of their genome and to share both haplotypes IBD for 25%. By varying the depth, we could see the change in the expected bias (Additional file 1: Fig S11) suggesting clearly that additional uncertainty or ‘fuzziness’ in the genotype likelihoods gives a stronger downward bias in a GRM moment-estimate. In Additional file 1: Fig S11, the average point-wise estimates of kinship are plotted against the varying values of ${\upsilon }^{i{i}^{{\prime}}j}\, {:=}\, \frac{1}{2}\left({\upsilon }^{ij}+{\upsilon }^{{i}^{^{\prime}}j}\right)$ where the indices $i$ and ${i}^{^{\prime}}$ denote the two siblings. Different mean values of ${\upsilon }^{i{i}^{^{\prime}}j}$ came from simulating read data with depths varying between 2 $\times$ and 25 $\times$. Here, we observed roughly linear relationships, with the slope depending on the minor allele frequency of the variant and with a slightly convex slope observed for the rarest variants. We can also see that as ${\upsilon }^{i{i}^{^{\prime}}j}$ tends to zero, the multiplicative bias in our estimate tends to one; and thus the estimator becomes unbiased. This suggests that if we can have a model for this relationship between bias and the fuzziness of each variant, it should be possible to gain an estimation of the unbiased value of the relatedness coefficients between $i$ and ${i}^{^{\prime}}$. Hence we used an idea similar to simulation extrapolation [46] though rather than artificially adding more noise to our data, we simply take advantage of the different levels of noise at different SNPs and extrapolate what our relatedness estimators would be with zero noise.

Our point wise estimates for the two matrices are written as ${\tilde{K }}_{i{i}^{^{\prime}}}^{j}$ and ${\tilde{D }}_{i{i}^{^{\prime}}}^{j}$ and we use linear regression to perform what was found to be the most successful modelling approach:

$$E\left[{K}_{i{i}^{^{\prime}}}^{j}\right]=\left({z}_{1}+{z}_{2}{\varphi }_{i{i}^{^{\prime}}}\right)\left(1+{z}_{3}\left({\upsilon }^{ij}+{\upsilon }^{{i}^{^{\prime}}j}\right)+{z}_{4}{\upsilon }^{ij}{\upsilon }^{{i}^{^{\prime}}j}\right)$$

$$E\left[{D}_{i{i}^{^{\prime}}}^{j}\right]=\left({u}_{1}+{u}_{2}{\psi }_{i{i}^{^{\prime}}}\right)\left(1+{u}_{3}\left({\upsilon }^{ij}+{\upsilon }^{{i}^{^{\prime}}j}\right)+{u}_{4}{\upsilon }^{ij}{\upsilon }^{{i}^{^{\prime}}j}\right)$$

This model, selected empirically based on its performance, allows for the pair of individuals to have different levels of uncertainty, and the interaction term ${\upsilon }^{ij}{\upsilon }^{{i}^{^{\prime}}j}$ may help to allow for the not completely linear relationships observed in Additional file 1: Fig. S11.

Here, ${\varphi }_{i{i}^{^{\prime}}}$ and ${\psi }_{i{i}^{^{\prime}}}$ are the kinship and fraternity coefficients between $i$ and ${i}^{^{\prime}}$, respectively. The model represents the intuition that when the fuzziness (${\upsilon }^{ij}$ and ${\upsilon }^{{i}^{^{\prime}}j}$) is null, the pointwise estimators should have expected values of the ‘true’ pointwise estimator from full WGS data; though we allow for the expectation to be linear in the ‘true’ estimator by introducing quantities ${z}_{1}$ and ${z}_{2}$ for kinship and ${u}_{1}$ and ${u}_{2}$ for fraternity. Indeed, all quantities ${z}_{1-4}$ and ${u}_{1-4}$ are nuisance parameters that allow a flexible modelling of potential biases that could be created by studying low-depth data.

Using this model, regressing values of ${\tilde{K }}_{i{i}^{^{\prime}}}^{j}$ or ${\tilde{D }}_{i{i}^{^{\prime}}}^{j}$ across values of $j$ against corresponding values of ${\upsilon }^{ij}$ and ${\upsilon }^{{i}^{^{\prime}}j}$ leads to estimates of ${\varphi }_{i{i}^{^{\prime}}}$ and ${\psi }_{i{i}^{^{\prime}}}$ from the intercepts of the linear regression models. Our adjustment procedure circumvents the nuisance parameters by firstly performing the aforementioned regression on the diagonal elements of the matrices $\tilde{K }$ and $\tilde{D }$ ($i={i}^{^{\prime}}$) with the knowledge that $2{\varphi }_{ii}$ and ${\psi }_{ii}$ should be equal to 1. Then in a second step, we regress the mean (unadjusted) estimates (${\tilde{K }}_{i{i}^{^{\prime}}}$ or ${\tilde{D }}_{i{i}^{^{\prime}}}$) against the intercepts from the aforementioned linear regression models that compared ${\tilde{K }}_{i{i}^{^{\prime}}}^{j}$ or ${\tilde{K }}_{i{i}^{^{\prime}}}^{j}$ with ${\upsilon }^{ij}$ and ${\upsilon }^{{i}^{^{\prime}}j}$ in order to calculate the appropriate multiplicative biases ${\beta }_{1}$ and ${\beta }_{2}$, thus providing the required adjustment of the initial estimates of LowKi.

This adjustment procedure carries a computational burden, so we apply it to only a subset of pairs which are chosen to represent a good range of relatedness estimates ($\widehat{\varphi }$ or $\widehat{\psi }$) among the unadjusted estimates in the sample calculated by LowKi. The adjustment procedure requires a set of pairs with variable unadjusted estimates to be successful, hence the default settings are to take the 20 pairs with the highest unadjusted estimates, the 20 pairs with the lowest unadjusted estimates, and all pairs involving a further random 100 individuals; but this can be changed by the user allowing for an adjustment using all pairs from the sample using the option $\tt adjust.par=c(0,0,n)$ where $n$ is the sample size of the dataset being analysed. The impact of the choice of these parameters is small but we did observe that certain choices could provide very poor estimates which we have protected against by restricting the possible choices that the user can provide; see Supplementary Materials section ‘Adjustment parameters’ and Additional file 1: Fig. S12 for more details.

After performing multiple tests of LowKi in different setting, we observed that in the case of small sample-sizes or when using externally sourced allele frequencies, the final estimates sometimes could be upwardly or downwardly biased by a small additive factor and all unrelated pairs would have coefficients distinct from zero. The final step of LowKi makes an adjustment by shifting the 25^th^ quartile of all coefficients to zero. This makes an assumption that for all intents and purposes of LowKi, a large proportion of unrelated pairs will be present in the sample (LowKi is aimed at larger samples), and hence this quartile will indicate an unrelated pair who should have coefficients close to zero.

### Testing existing software

To run SEEKIN (v1.01), we first applied BEAGLE (v4.1). BEAGLE was given reference haplotypes from the 1000 Genomes project (Phase 3) and was run in windows of 750 variants with buffers of 250 variants. We found that BEAGLE required very long runtimes, hence we set the parameter ‘modelscale’ equal to 3 which the authors of BEAGLE suggested in the software’s manual as an appropriate setting to increase both speed and accuracy when applying BEAGLE to genotype likelihood data. Otherwise, both NGSRelateV2 and SEEKIN were run with the default recommended parameters.

### Testing on real data

In order to test our method on a real dataset, we were given access to 150 individuals from FranceGenRef and down-sampled their individual bam files to an average of 2.5× coverage. The FranceGenRef panel comprises 856 individuals from the population of France and combines individuals from the GAZEL cohort (www.gazel.inserm.fr/en), from the PREGO cohort (www.vacarme-project.org), and 50 blood donors from the Finistere region. The down-sampling was achieved by simply counting the number of reads in the original bam-files, and randomly sampling the appropriate proportion of these reads given that full bam files correspond to average read depth of 35×. This set of 150 individuals contains two sibling pairs who have an expected kinship of 0.25 and expected fraternity coefficient of 0.25. All other pairs are expected to have kinship and fraternity coefficients very close to zero. There may be residual population structure in the sample as individuals of FranceGenRef come from different regions in France; a country with substantial fine-scale population structure [47]. Down-sampling and calling were performed with samtools (v0.1.19) [48], Sambamba (v0.7.1) [49], and GATK HaplotyeCaller (v3.7) which provides the genotype likelihoods that we supplied to LowKi as well as NGSRelateV2, and BEAGLE followed by SEEKIN.

We observed that LowKi’s estimators were improved if variants with a very small observed expected minor allele frequency were removed from the calculation and such a filter has been added as a default option in LowKi. Specifically, the quantity ${\overline{P}}_{Aa}^{j}+2{\overline{P}}_{aa}^{j}$ should be in the range 0.05 to 1.95. In the example of the 150 individuals of FranceGenRef, 1,009,181 variants out of a possible 1,051,789 were used in the calculation and the same variant set was also provided to NGSRelateV2 for comparability.

### Testing LowKi in diverse settings

To further explore the performance of LowKi in other sequencing scenarios, we constructed simulated datasets based on data from the 1000 Genomes Project using the R-package Mozza (https://github.com/genostats/Mozza). Mozza simulates multiple generations, building haplotype mosaics for generation $n$ based on haplotypes present in generation $n-1$. Beginning with haplotypes built as a mosaic (the tiles of which having exponentially distributed length with expectation 20 cM) of the European haplotypes from the 1000 Genomes project, 5 generations of 1000 individuals were simulated and 200 individuals from the last two generations were retained, ensuring the presence of related individuals. Using different realisations of this process of simulating such groups of 200 individuals, we simulated different sequencing scenarios of samples including related individuals (with sizable kinship and fraternity coefficients). In this way we tested LowKi and NGSRelateV2 at very low depths, in samples with heterogeneity in their sequencing depth, and for very small sample sizes.

## Supplementary Information

**Additional file 1**. Supplementary material and figures.

## Data Availability

LowKi is freely available at https://github.com/genostats/LowKi and implemented in R. Instructions for download and implementation as well as example datasets are also provided at this location. The package contains a small simulated dataset allowing to test the method. Contact for applications for access to genetic data from the Cilento isolates: Marina Ciullo (marina.ciullo@igb.cnr.it). Contacts for applications for access to simulation datasets based on the pedigree structure of the Cilento Isolates: Marina Ciullo (marina.ciullo@igb.cnr.it), Anne-Louise Leutenegger (anne-louise.leutenegger@inserm.fr) and Anthony F. Herzig (anthony.herzig@inserm.fr). The FranceGenRef panel data will be submitted by the FranceGenRef consortium to the French Centralized Data Center of the France Medicine Genomic Plan that is under construction. Enquiries for the use of this data can be addressed to GENMED LABEX (http://www.genmed.fr/index.php/en/contact).

## References

[1] Novembre J, Johnson T, Bryc K, Kutalik Z, Boyko AR, Auton A (2008). Genes mirror geography within Europe. Nature.

[2] Menozzi P, Piazza A, Cavalli-Sforza L (1978). Synthetic maps of human gene frequencies in Europeans. Science.

[3] Price AL, Patterson NJ, Plenge RM, Weinblatt ME, Shadick NA, Reich D (2006). Principal components analysis corrects for stratification in genome-wide association studies. Nat Genet.

[4] Price AL, Zaitlen NA, Reich D, Patterson N (2010). New approaches to population stratification in genome-wide association studies. Nat Rev Genet.

[5] Yang J, Benyamin B, McEvoy BP, Gordon S, Henders AK, Nyholt DR (2010). Common SNPs explain a large proportion of the heritability for human height. Nat Genet.

[6] Yang J, Lee SH, Goddard ME, Visscher PM (2011). GCTA: a tool for genome-wide complex trait analysis. Am J Hum Genet.

[7] Zhu Z, Bakshi A, Vinkhuyzen AAE, Hemani G, Lee SH, Nolte IM (2015). Dominance genetic variation contributes little to the missing heritability for human complex traits. Am J Hum Genet.

[8] Herzig AF, Nutile T, Ruggiero D, Ciullo M, Perdry H, Leutenegger A-L. Detecting the dominance component of heritability in isolated and outbred human populations. Sci Rep. 2018;8(1). 10.1038/s41598-018-36050-7 PMC6303332 30575761

[9] Speed D, Balding DJ (2015). Relatedness in the post-genomic era: is it still useful?. Nat Rev Genet.

[10] Thompson EA (2013). Identity by descent: variation in meiosis, across genomes, and in populations. Genetics.

[11] Weir BS, Anderson AD, Hepler AB (2006). Genetic relatedness analysis: modern data and new challenges. Nat Rev Genet.

[12] Goudet J, Kay T, Weir BS (2018). How to estimate kinship. Mol Ecol.

[13] Sims D, Sudbery I, Ilott NE, Heger A, Ponting CP (2014). Sequencing depth and coverage: key considerations in genomic analyses. Nat Rev Genet.

[14] Gilly A, Ritchie GR, Southam L, Farmaki A-E, Tsafantakis E, Dedoussis G (2016). Very low-depth sequencing in a founder population identifies a cardioprotective APOC3 signal missed by genome-wide imputation. Hum Mol Genet.

[15] Converge Consortium, Cai N, Bigdeli TB, Kretzschmar W, Li Y, Liang J, et al. Sparse whole-genome sequencing identifies two loci for major depressive disorder. Nature. 2015;523(7562):588–91. 10.1038/nature14659 PMC4522619 26176920

[16] the Haplotype Reference Consortium, McCarthy S, Das S, Kretzschmar W, Delaneau O, Wood AR, et al. A reference panel of 64,976 haplotypes for genotype imputation. Nature Genetics. 2016;48(10):1279–83. 10.1038/ng.3643 PMC5388176 27548312

[17] Hofreiter M, Paijmans JLA, Goodchild H, Speller CF, Barlow A, Fortes GG (2015). The future of ancient DNA: technical advances and conceptual shifts. BioEssays.

[18] Städele V, Vigilant L (2016). Strategies for determining kinship in wild populations using genetic data. Ecol Evol.

[19] Vieira FG, Fumagalli M, Albrechtsen A, Nielsen R (2013). Estimating inbreeding coefficients from NGS data: impact on genotype calling and allele frequency estimation. Genome Res.

[20] Hanghøj K, Moltke I, Andersen PA, Manica A, Korneliussen TS. Fast and accurate relatedness estimation from high-throughput sequencing data in the presence of inbreeding. Gigascience. 2019;8(5). 10.1093/gigascience/giz034 PMC6488770 31042285

[21] Korneliussen TS, Moltke I (2015). NgsRelate: a software tool for estimating pairwise relatedness from next-generation sequencing data. Bioinformatics.

[22] Lipatov M, Sanjeev K, Patro R, Veeramah KR. Maximum likelihood estimation of biological relatedness from low coverage sequencing data. bioRxiv. 2015;023374.

[23] Dou J, Sun B, Sim X, Hughes JD, Reilly DF, Tai ES (2017). Estimation of kinship coefficient in structured and admixed populations using sparse sequencing data. PLoS Genet.

[24] Perdry H, Dandine-Rolland C, Banddyopadhyay D, Kettner L. Gaston: Genetic data handling (QC, GRM, LD, PCA) & linear mixed models. CRAN. 2018;https://cran.r-project.org/web/packages/gaston/index.html.

[25] Colonna V, Nutile T, Astore M, Guardiola O, Antoniol G, Ciullo M (2007). Campora: a young genetic isolate in South Italy. Hum Hered.

[26] Colonna V, Nutile T, Ferrucci RR, Fardella G, Aversano M, Barbujani G (2009). Comparing population structure as inferred from genealogical versus genetic information. Eur J Hum Genet.

[27] Nutile T, Ruggiero D, Herzig AF, Tirozzi A, Nappo S, Sorice R, et al. Whole-exome sequencing in the isolated populations of Cilento from South Italy. Sci Rep. 2019;9(1). 10.1038/s41598-019-41022-6 PMC6411969 30858532

[28] Herzig AF, Nutile T, Babron M-C, Ciullo M, Bellenguez C, Leutenegger A-L (2018). Strategies for phasing and imputation in a population isolate. Genetic Epidemiol.

[29] Alves I, Giemza J, Blum M, Bernhardsson C, Chatel S, Karakachoff M, et al. Genetic population structure across Brittany and the downstream Loire basin provides new insights on the demographic history of Western Europe. bioRxiv. 2022;478491.

[30] Herzig AF, Velo-Suárez L, Frex Consortium, FranceGenRef Consortium, Dina C, Redon R, et al. Can imputation in a European country be improved by local reference panels? The example of France. bioRxiv. 2022;480829.

[31] The 1000 Genomes Project Consortium. A global reference for human genetic variation. Nature. 2015;526(7571):68–74. 10.1038/nature15393 PMC4750478 26432245

[32] Browning BL, Browning SR (2016). Genotype imputation with millions of reference samples. Am J Hum Genet.

[33] Korneliussen TS, Albrechtsen A, Nielsen R (2014). ANGSD: analysis of next generation sequencing data. BMC Bioinform.

[34] Kim SY, Lohmueller KE, Albrechtsen A, Li Y, Korneliussen T, Tian G (2011). Estimation of allele frequency and association mapping using next-generation sequencing data. BMC Bioinform.

[35] DePristo MA, Banks E, Poplin R, Garimella KV, Maguire JR, Hartl C (2011). A framework for variation discovery and genotyping using next-generation DNA sequencing data. Nat Genet.

[36] McKenna A, Hanna M, Banks E, Sivachenko A, Cibulskis K, Kernytsky A (2010). The Genome Analysis Toolkit: a MapReduce framework for analyzing next-generation DNA sequencing data. Genome Res.

[37] Van der Auwera GA, Carneiro MO, Hartl C, Poplin R, Del Angel G, Levy-Moonshine A, et al. From FastQ data to high confidence variant calls: the Genome Analysis Toolkit best practices pipeline. Curr Protoc Bioinformatics. 2013;43:11.10.1–33. 10.1002/0471250953.bi1110s43 PMC4243306 25431634

[38] Muyas F, Bosio M, Puig A, Susak H, Domènech L, Escaramis G (2019). Allele balance bias identifies systematic genotyping errors and false disease associations. Hum Mutat.

[39] Li H (2014). Toward better understanding of artifacts in variant calling from high-coverage samples. Bioinformatics.

[40] The UK10K Consortium, Walter K, Min JL, Huang J, Crooks L, Memari Y, et al. The UK10K project identifies rare variants in health and disease. Nature. 2015;526:82. 10.1038/nature14962 PMC4773891 26367797

[41] Wijsman EM, Rothstein JH, Thompson EA (2006). Multipoint linkage analysis with many multiallelic or dense diallelic markers: Markov chain-Monte Carlo provides practical approaches for genome scans on general pedigrees. Am J Hum Genet.

[42] Fisher RA (1919). XV—The correlation between relatives on the supposition of Mendelian inheritance. Earth Environ Sci Trans R Soc Edinb.

[43] Vitezica ZG, Legarra A, Toro MA, Varona L (2017). Orthogonal estimates of variances for additive, dominance, and epistatic effects in populations. Genetics.

[44] VanRaden PM (2007). Genomic measures of relationship and inbreeding. Interbull Annu Meet Proc.

[45] VanRaden PM (2008). Efficient methods to compute genomic predictions. J Dairy Sci.

[46] Cook JR, Stefanski LA (1994). Simulation-extrapolation estimation in parametric measurement error models. J Am Stat Assoc.

[47] Saint Pierre A, Giemza J, Alves I, Karakachoff M, Gaudin M, Amouyel P (2020). The genetic history of France. Eur J Hum Genet.

[48] Li H, Handsaker B, Wysoker A, Fennell T, Ruan J, Homer N (2009). The Sequence Alignment/Map format and SAMtools. Bioinformatics.

[49] Tarasov A, Vilella AJ, Cuppen E, Nijman IJ, Prins P (2015). Sambamba: fast processing of NGS alignment formats. Bioinformatics.

