# Supplementary material for: Moment estimators of relatedness from low-depth whole-genome sequencing data
Source: BMC Bioinformatics. 2022 Jun 24;23:254. doi: 10.1186/s12859-022-04795-8 (PMC9233360; doi:10.1186/s12859-022-04795-8)
Supplement: Supplementary file 1 — Additional file 1. Supplementary material and figures. [file 12859_2022_4795_MOESM1_ESM.docx]

**Moment estimators of relatedness from low-depth whole-genome sequencing data**

Herzig AF^1^, Ciullo M^2,3^, FranceGenRef Consortium^4^, Leutenegger A-L^5^, Perdry H^6^

1. Inserm, Univ Brest, EFS, UMR 1078, GGB, F-29200 Brest, France

2. Institute of Genetics and Biophysics A. Buzzati-Traverso - CNR, Naples, Italy

3. IRCCS Neuromed, Pozzilli, Isernia, Italy

4. LABEX GENMED, Centre National de Recherche en Génomique Humaine, Evry, Paris

5. Inserm, Université Paris Cité, UMR 1141, NeuroDiderot, F-75019 Paris, France

6. CESP Inserm U1018, Université Paris-Saclay, Villejuif, France

Corresponding Author:

Anthony Francis Herzig

anthony.herzig@inserm.fr

+33298017361

Inserm UMR 1078, 22 Avenue Camille Desmoulins, 29238 Brest, France

Supplementary Material and Figures

| 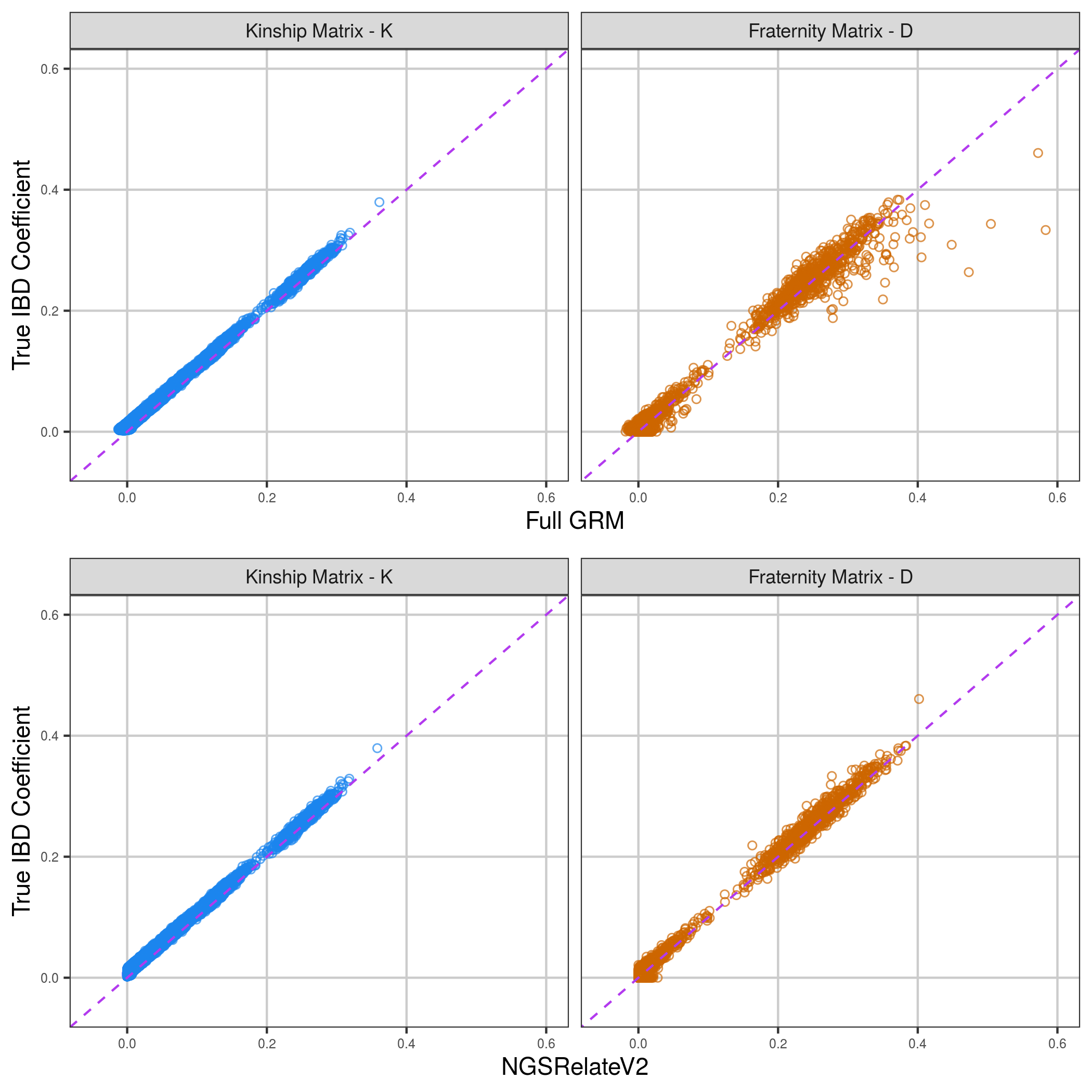 |
| --- |
| **Supplementary Figure S1a**  (Top) Full GRM off-diagonal elements against simulated IBD-sharing proportions in the CilentoSim population for the kinship and fraternity matrices, $K$and $D$.  (Bottom) Comparison of NGSRelateV2 estimates of kinship and fraternity based on simulated 10× data for the same group of individuals against the simulated IBD-sharing proportions.  From this figure, we observe that Full GRM estimates and the True IBD Coefficients are not in complete agreement. NGSRelateV2 approximates the True IBD Coefficients very well and so is a meaningful benchmark for NGSRelateV2, if we were to benchmark it against Full GRM estimates it would appear to be less accurate. However as LowKi is a moment estimate, which are seen here to have their limits in estimating IBD sharing, the best case scenario for LowKi would be to reproduce the estimates of moment estimators that have access to perfect genotype data; thus LowKi is benchmarked against Full GRM estimates. |


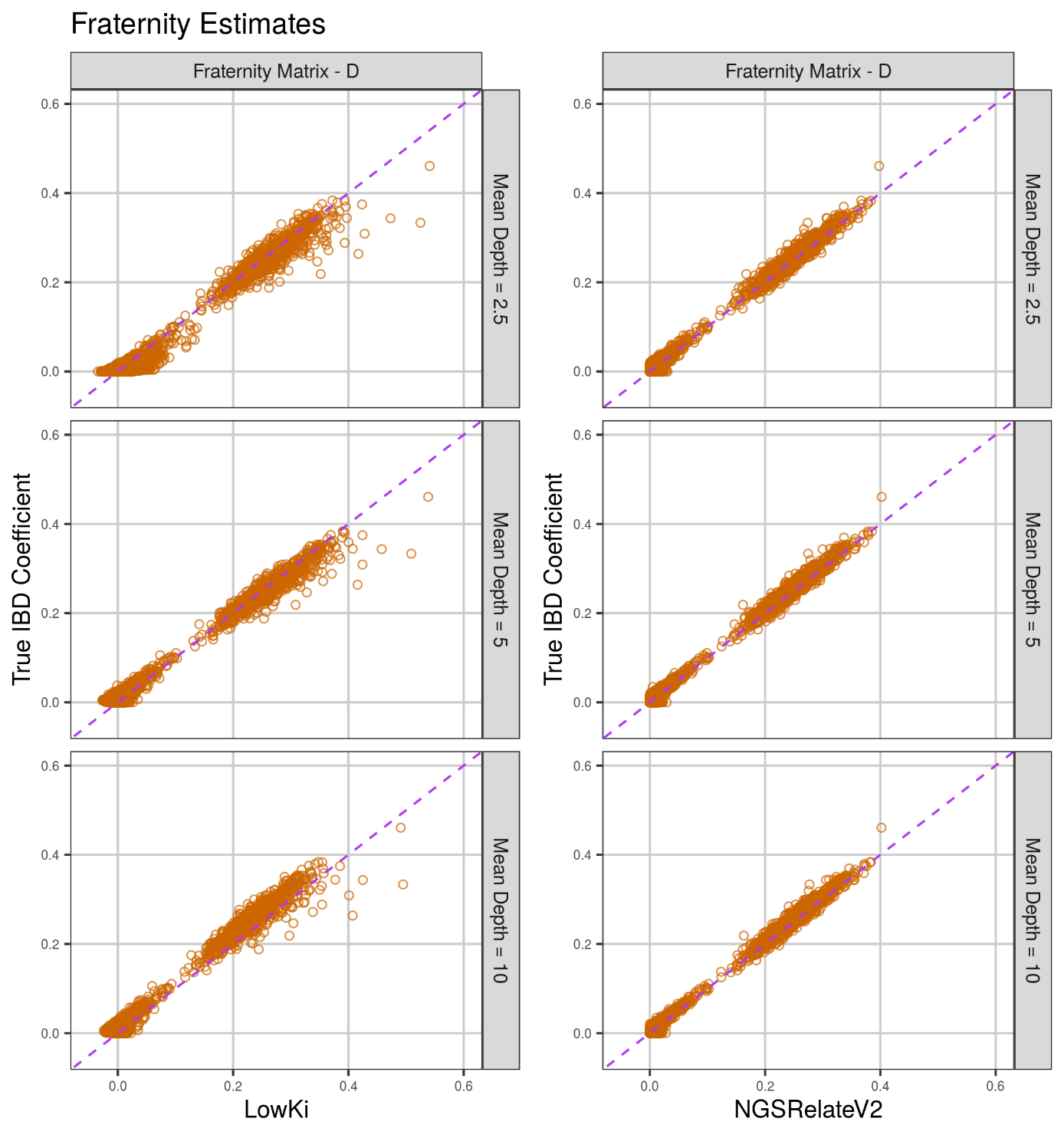


**Supplementary Figure S1b**

Estimates of fraternity in the CilentoSim dataset from LowKi and NGSRelateV2 compared to the True IBD Coefficients (these are known as they were recorded during the simulation of the data).


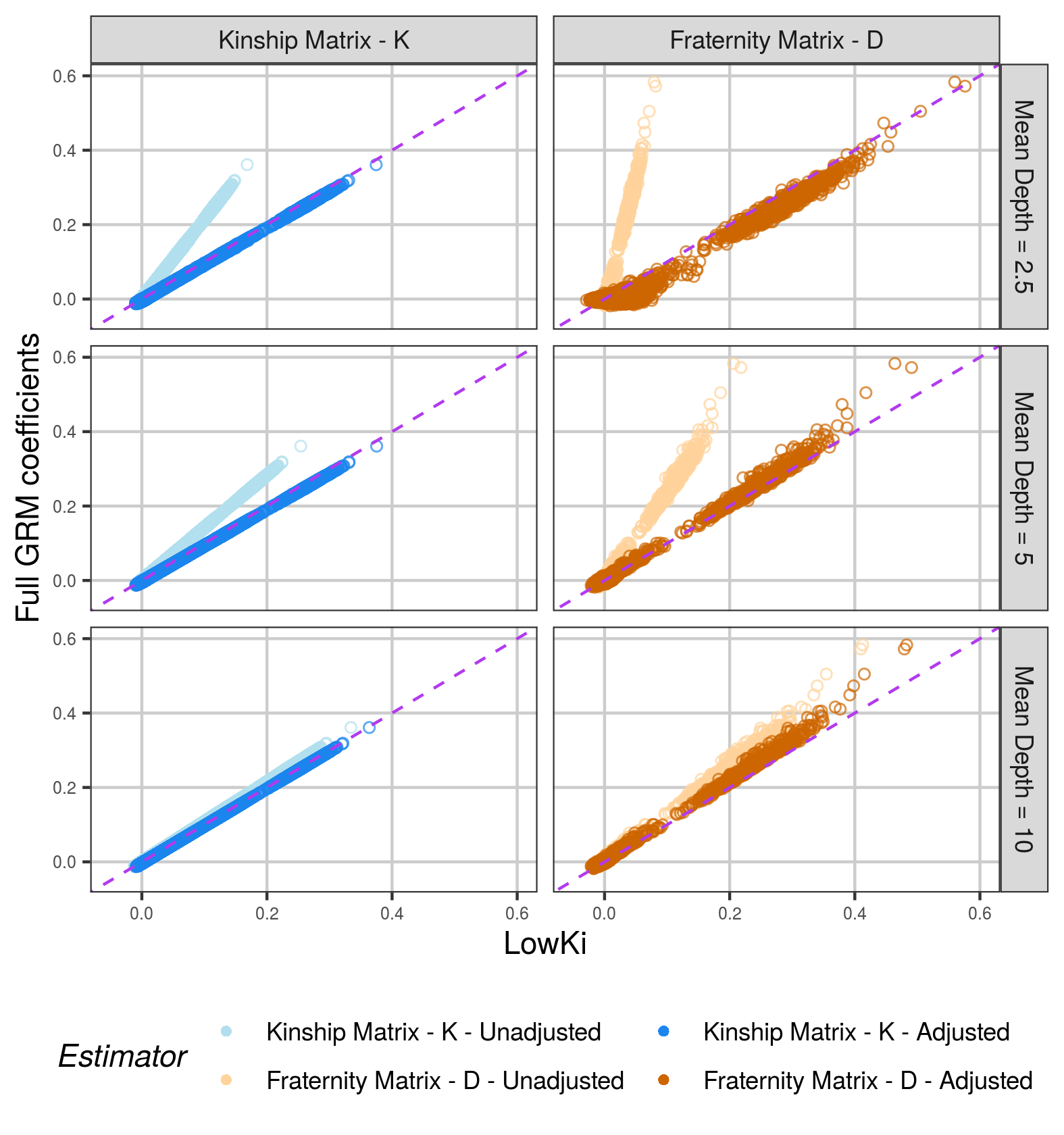


**Supplementary Figure S2**

LowKi’s ‘unadjusted’ and ‘adjusted’ estimates for kinship and fraternity for the CilentoSim dataset at simulated depths of 2.5$\times$, 5$\times$, and 10$\times$. Unadjusted estimates are the raw moment estimates calculated as GRMs based on genotype likelihoods described in the Methods in the main text. Adjusted estimates are the unadjusted estimated scaled by a multiplicative factor that is attained through LowKi’s regression based bias correction procedure; equally described in the Methods in the main text. As depth increases, the bias in the unadjusted estimates decreases.


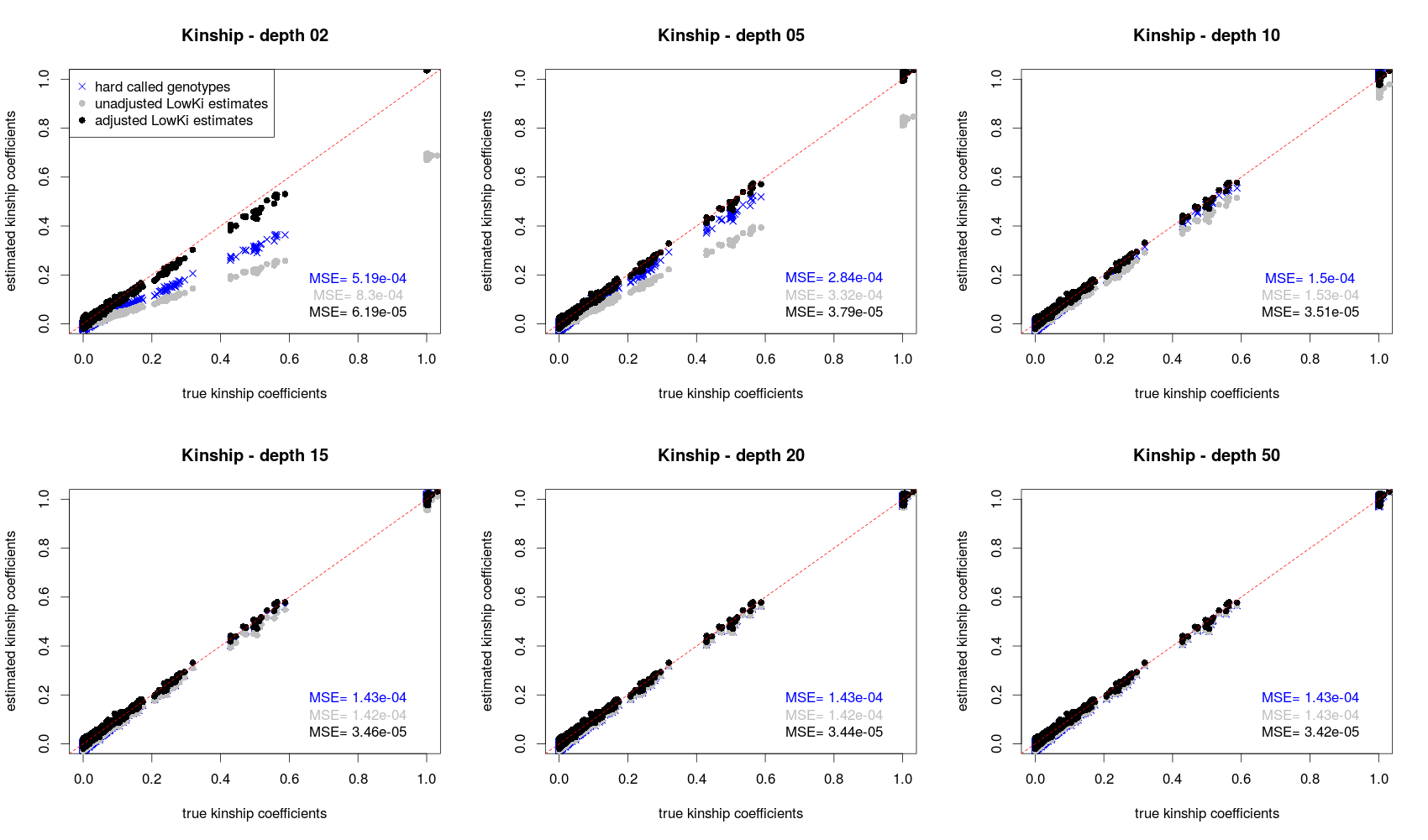


Supplementary Figure S3a


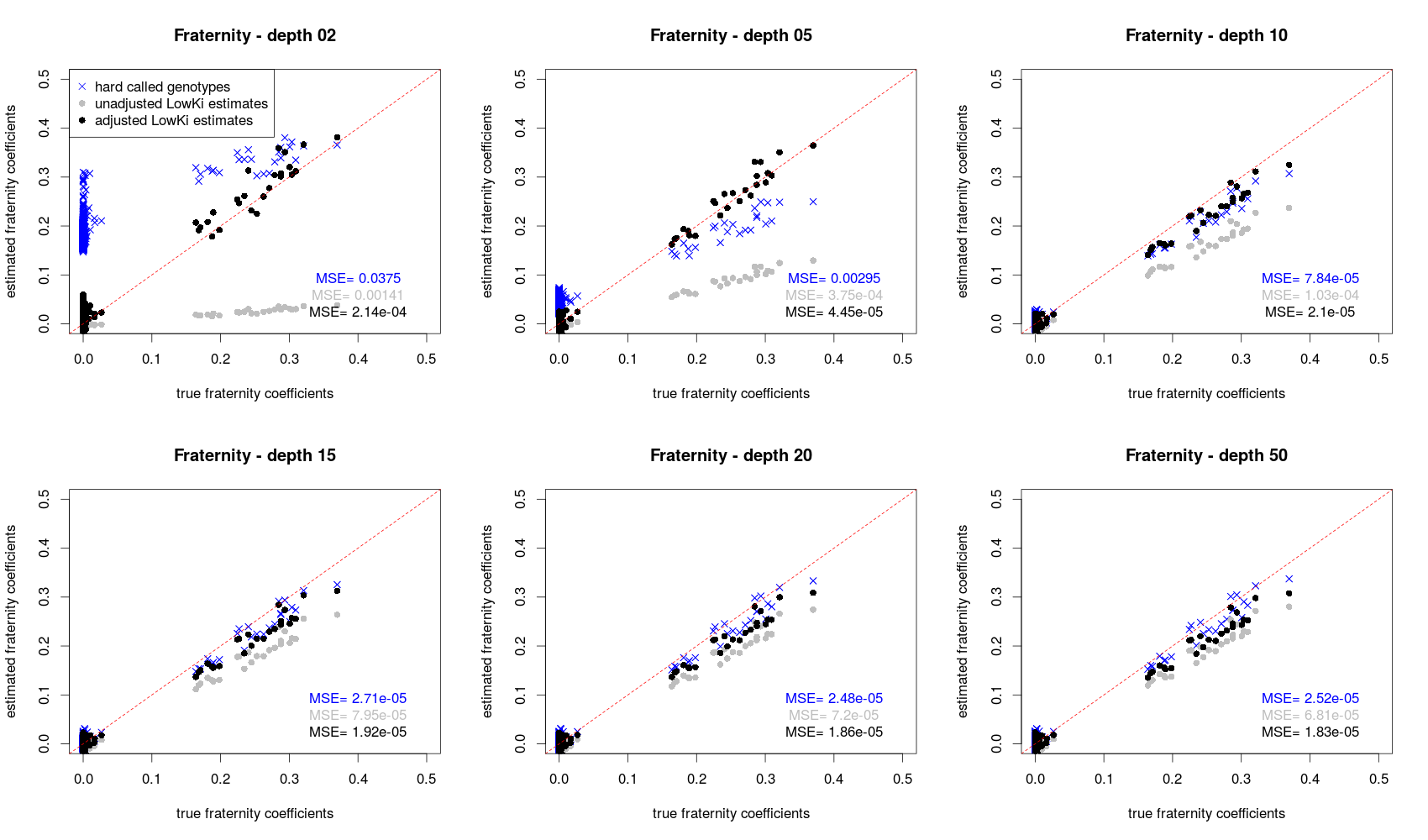


Supplementary Figure S3b

**Supplementary Figure S3a-b**

Having observed that LowKi appeared to underestimate fraternity in the CilentoSim dataset (see main text), we performed additional simulations to explore this observation. Presented here is an application of LowKi on 200 simulated individuals with 200,000 simulated SNPs created using Mozza and the European haplotypes of the 1000 Genomes Project. Individuals are simulated at depths 2$\times$, 5$\times$, 10$\times$, 15$\times$, 20$\times$, and 50$\times$. Results are compared to GMRs estimated using hard-called genotypes and to LowKi’s unadjusted estimates. The benchmarks are the true coefficients which are determined during the simulation. Indeed, for high depths LowKi may underestimate fraternity - but kinship remains well estimated. However, at such high depths it may not be necessary to estimate GRMs from genotype likelihoods as GMRs from hard-called genotypes become acceptable for depths above 10$\times$.

| 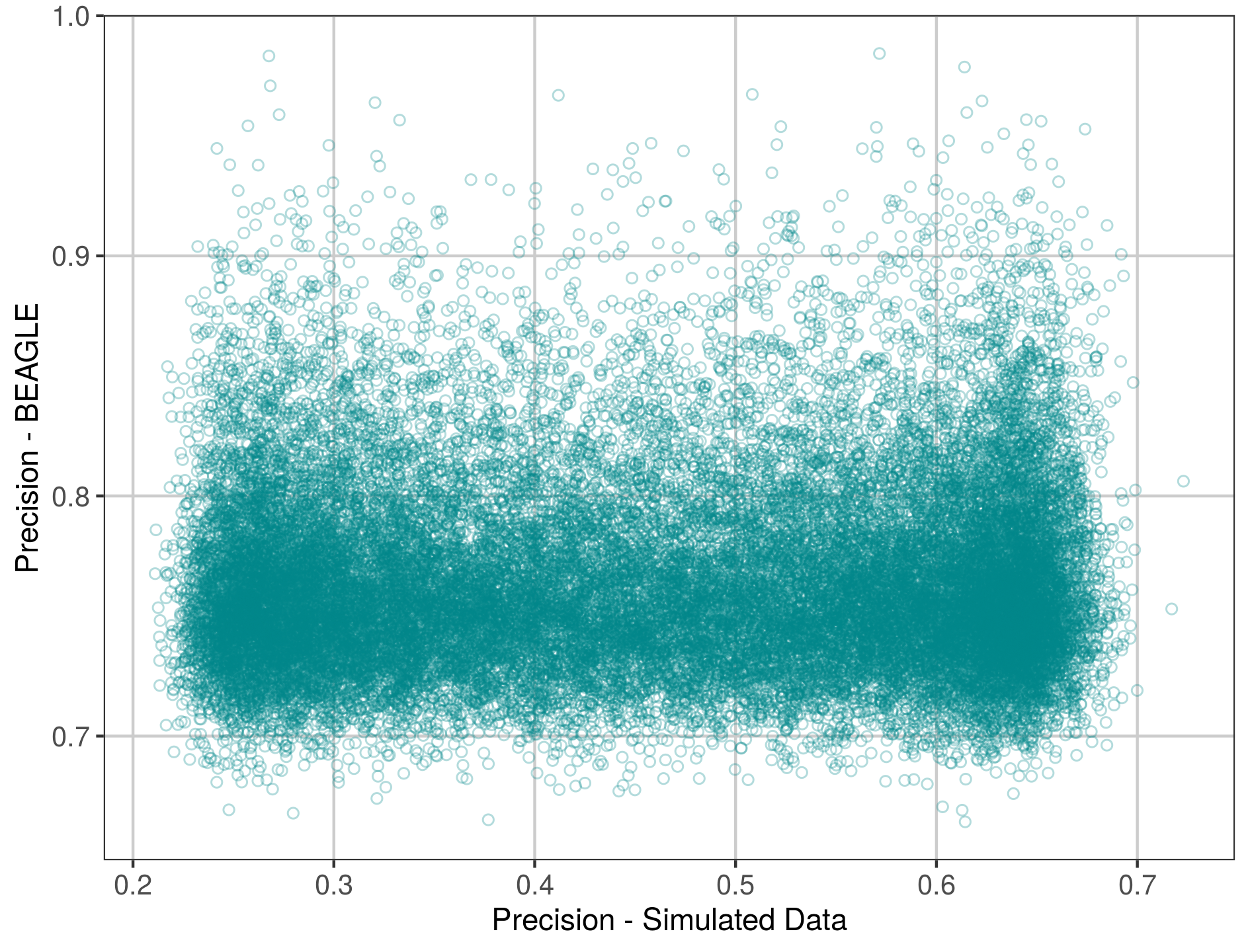 |
| --- |
| **Supplementary Figure S4**  Comparison of observed precision of three genotype probabilities for 25,000 variants. A values of precision of 1 indicates a set of probabilities such as (1,0,0) whereas a precision of 0 indicates probabilities $(\frac{1}{3},\frac{1}{3},\frac{1}{3})$. Precision was first calculated on the simulated genotypes likelihoods of CilentoSim at a depth of 2.5$\times$ (x-axis). Almost all precision statistics lie between 0.2 and 0.7. On the y-axis, the precision is given after BEAGLE has been applied. The resulting precision statistics are roughly between 0.7 and 1.0, demonstrating how SEEKIN benefits greatly from the improvements that BEAGLE makes to the raw data. |

*
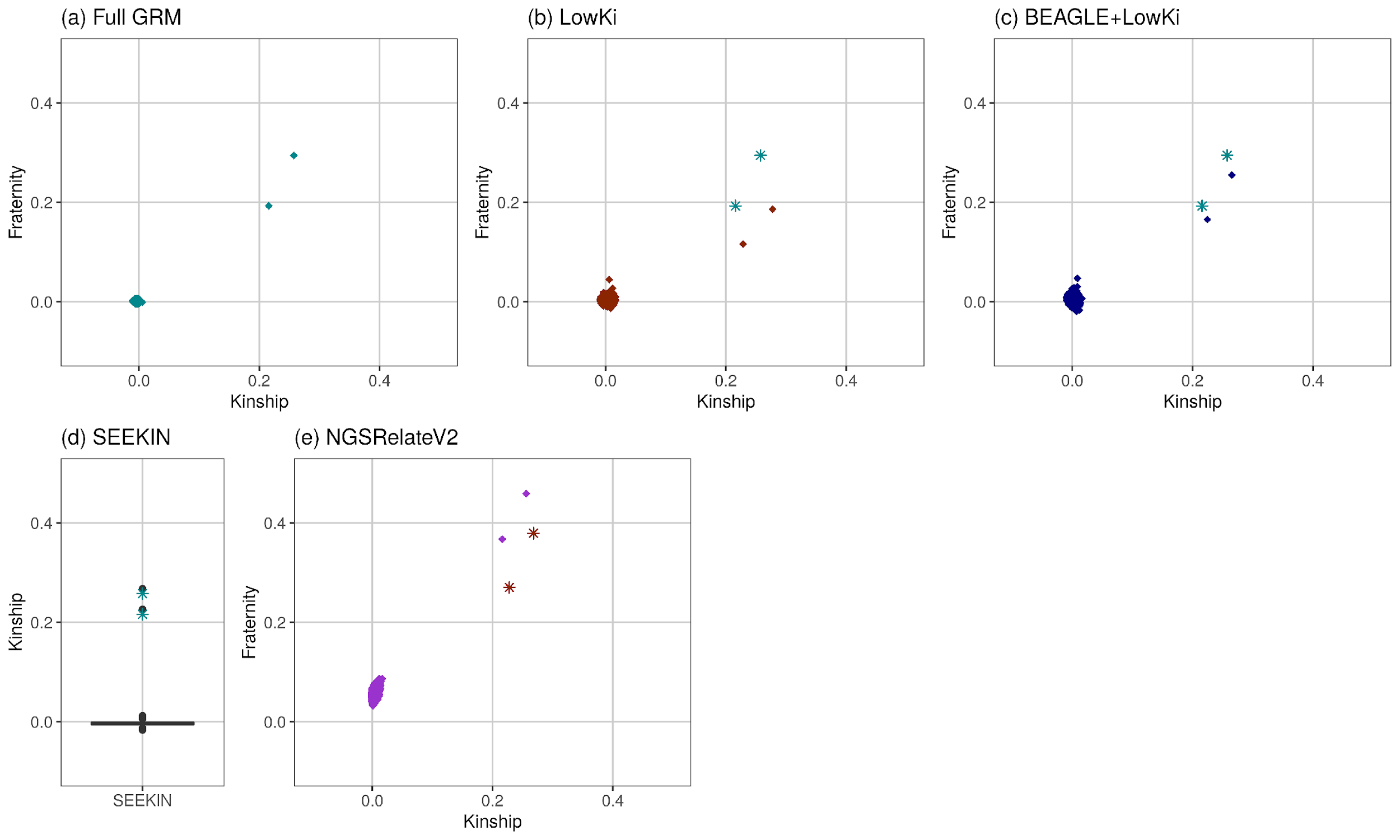
*

|  |
| --- |
| **Supplementary Figure S5**  Analysis of 150 individuals from the FranceGenRef panel including two sibling pairs, all methods could identify the two sibling pairs as clear outliers successfully.  Panel (a) - Kinship and Fraternity coefficients estimated by LowKi on genotypes (Full GRM) from the original 30-40$\times$ data, the two sibling pairs are visible.  Panel (b) - In dark red, the estimates of kinship and fraternity from down-sampled (2.5$\times$) data using our proposed moment estimators. The two stars represent the benchmark for these estimates coming from panel (a).  Panel (c) - Similar to panel (b) but here BEAGLE was used before applying our moment estimators in Gaston - a slight improvement in the estimation of fraternity was observed.  Panel (d) - Kinship estimates only from SEEKIN are given in a boxplot as SEEKIN does not estimate fraternity. Again, blue stars give the benchmark from the estimates of panel (a).  Panel (e) - In purple, the estimates of kinship and fraternity by NGSRelateV2 are given. Here the benchmark (dark red stars) are the estimated from NGSRelateV2 applied to the original 30-40$\times$ data. |


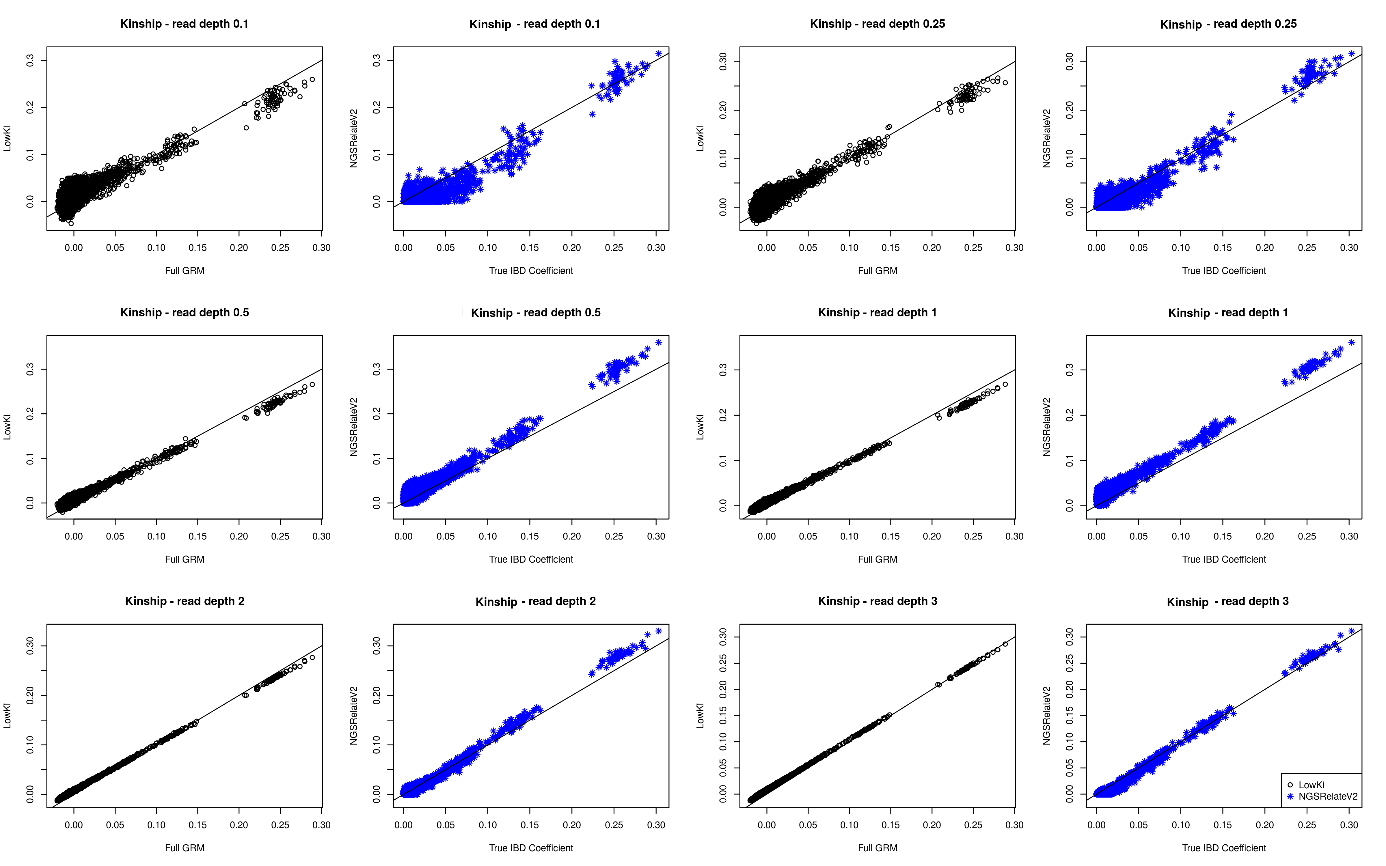


**Supplementary Figure S6**  Estimates of kinship from LowKi and NGSRelateV2 for very low-depth data, details follow below Supplementary Figure S7.


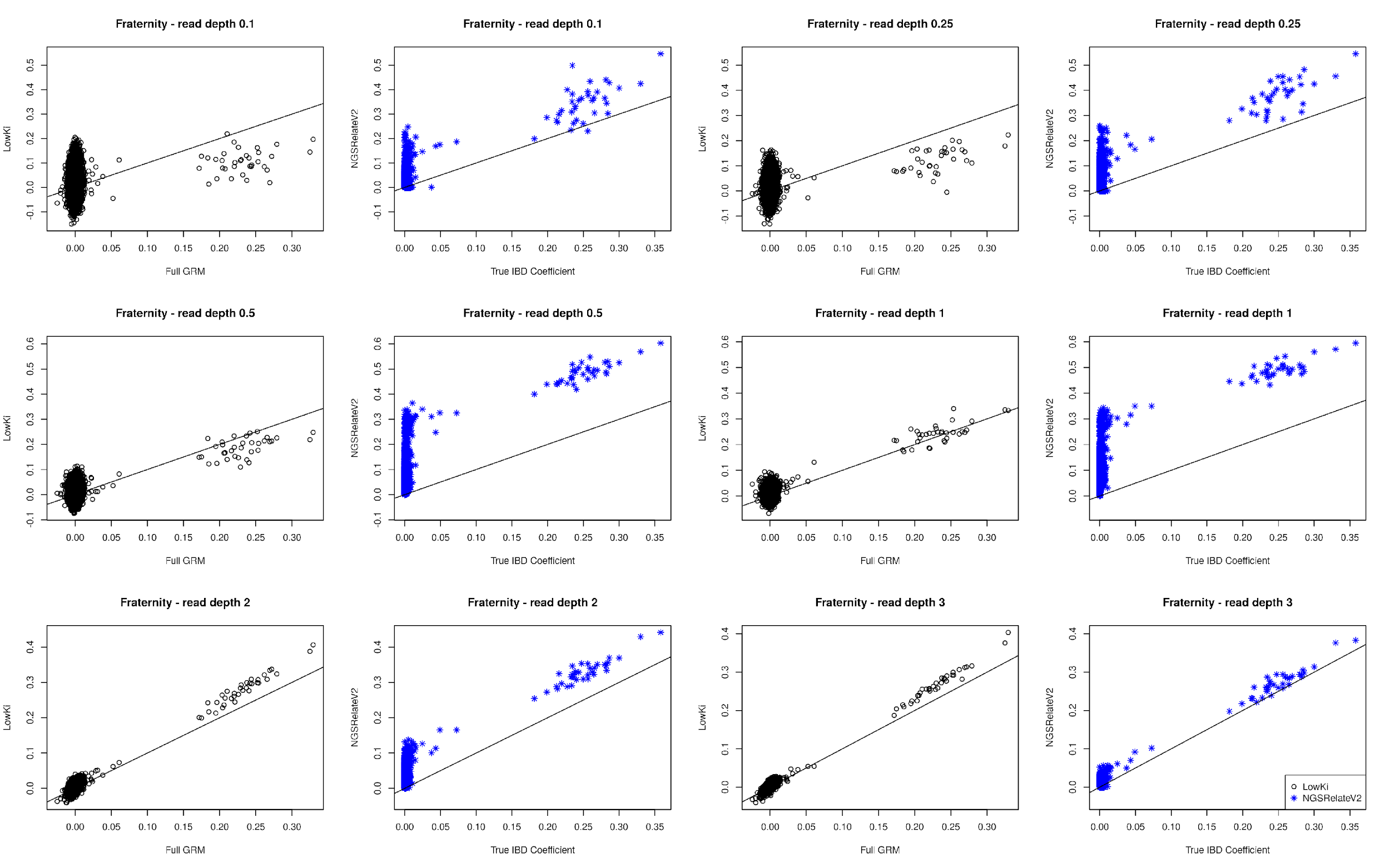


**Supplementary Figure S7**  Estimates of fraternity from LowKi and NGSRelateV2 for very low-depth data, details follow.

**Supplementary Figure S6 and S7**

Application of LowKi (black) and NGSRelateV2 (blue) to data with very low simulated sequencing depths. Simulated data was constructed using Mozza and the European haplotypes of the 1000 Genomes Project. 200 individuals with 200,000 SNPs were simulated with mean read depths of 0.1×, 0.25×, 0.5×, 1×, 2×, and 3×. As with the major analyses in the main text, we benchmark LowKi against Full GRM estimates (using simulated genotypes rather than genotype likelihoods) as this is represents the best possible case for a moment estimator; NGSRelateV2 is benchmarked against True IBD coefficients as this is a more meaningful comparison as had been observed in Supplementary Figure 1.


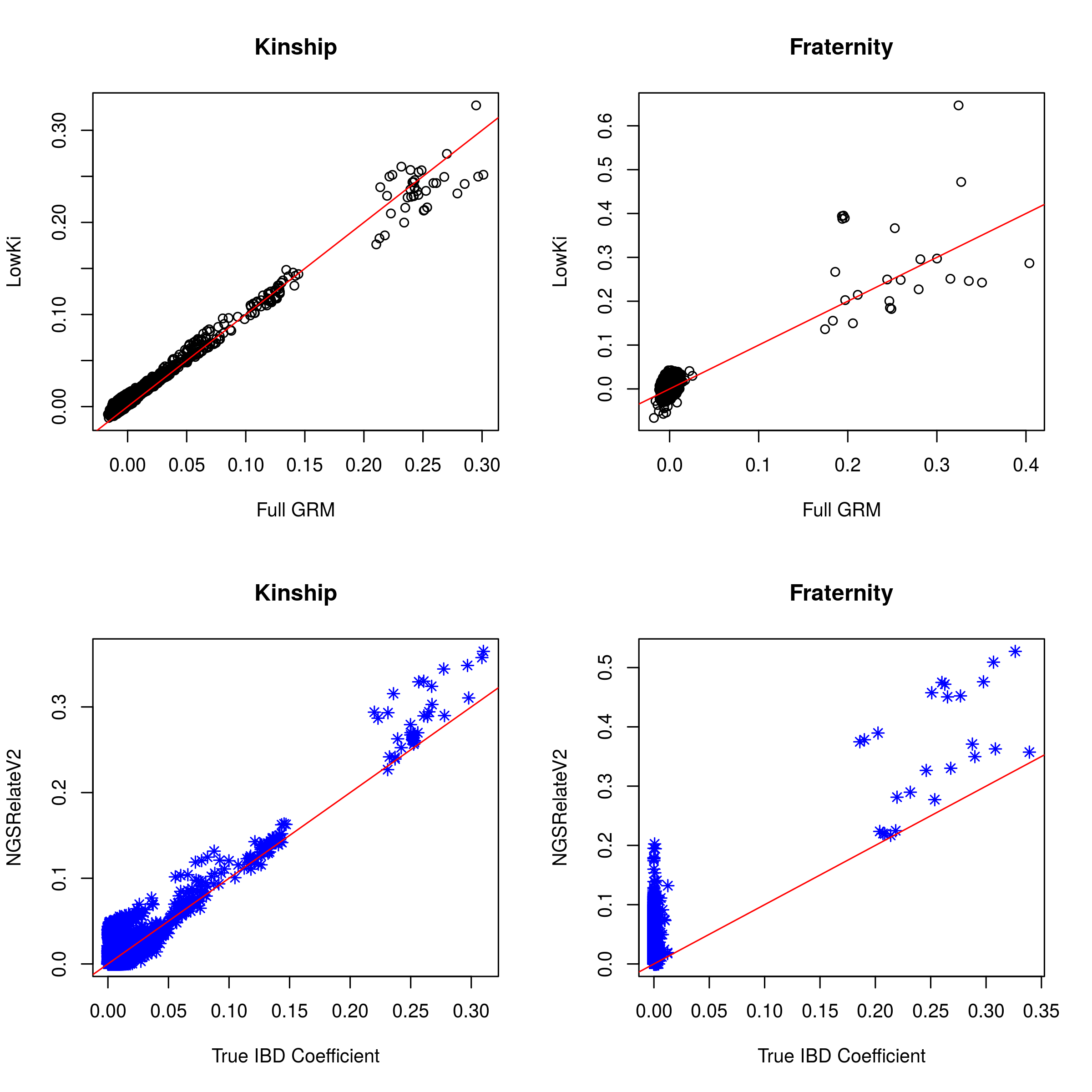


**Supplementary Figure S8**

Application of LowKi and NGSRelateV2 on 200 simulated individuals with per-individual heterogeneity of simulated sequencing depth across 200,000 simulated SNPs using Mozza and the European haplotypes of the 1000 Genomes Project. In this sample there are 4 groups of 50 individuals with mean sequencing depths of 2×, 3×, 4×, and 5×.


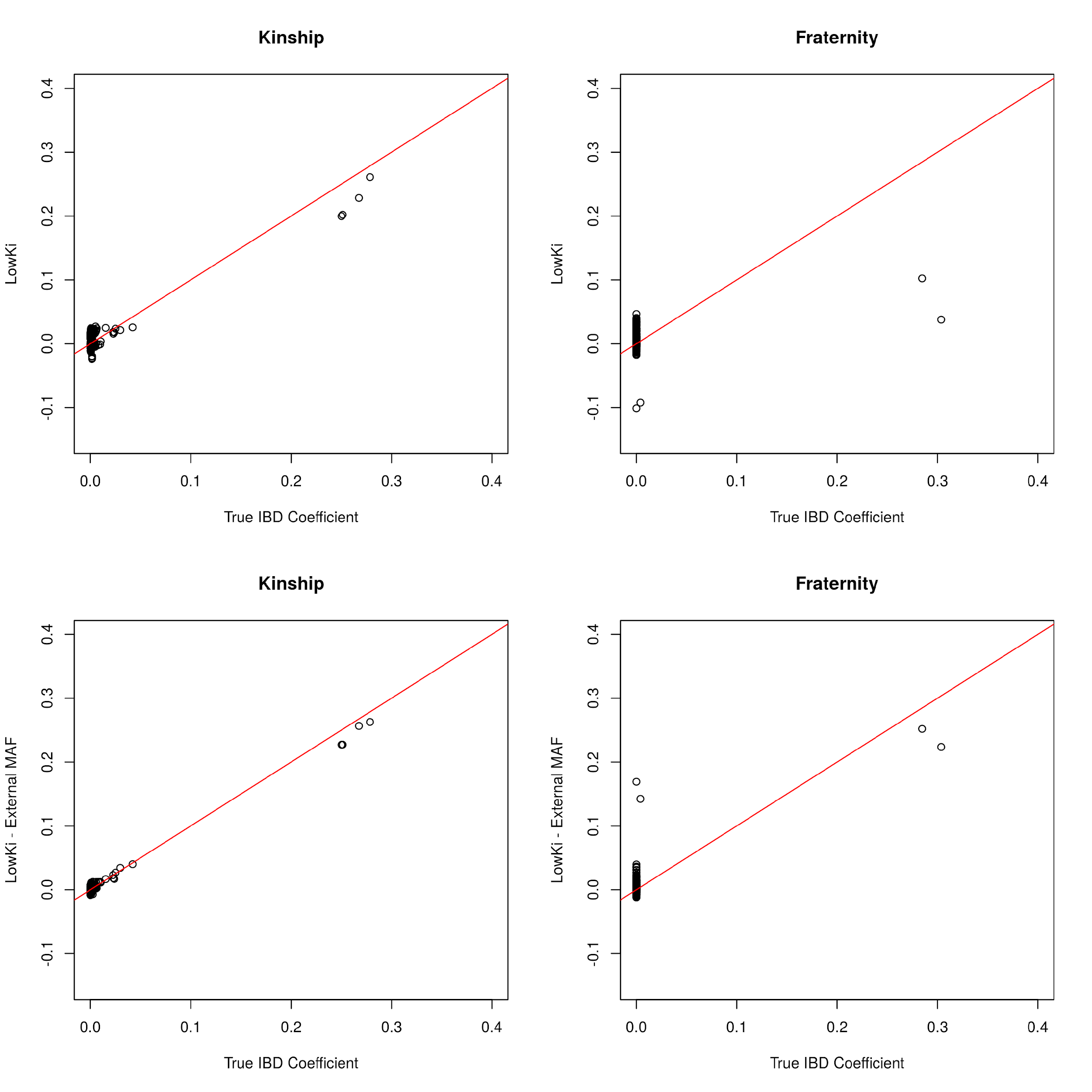

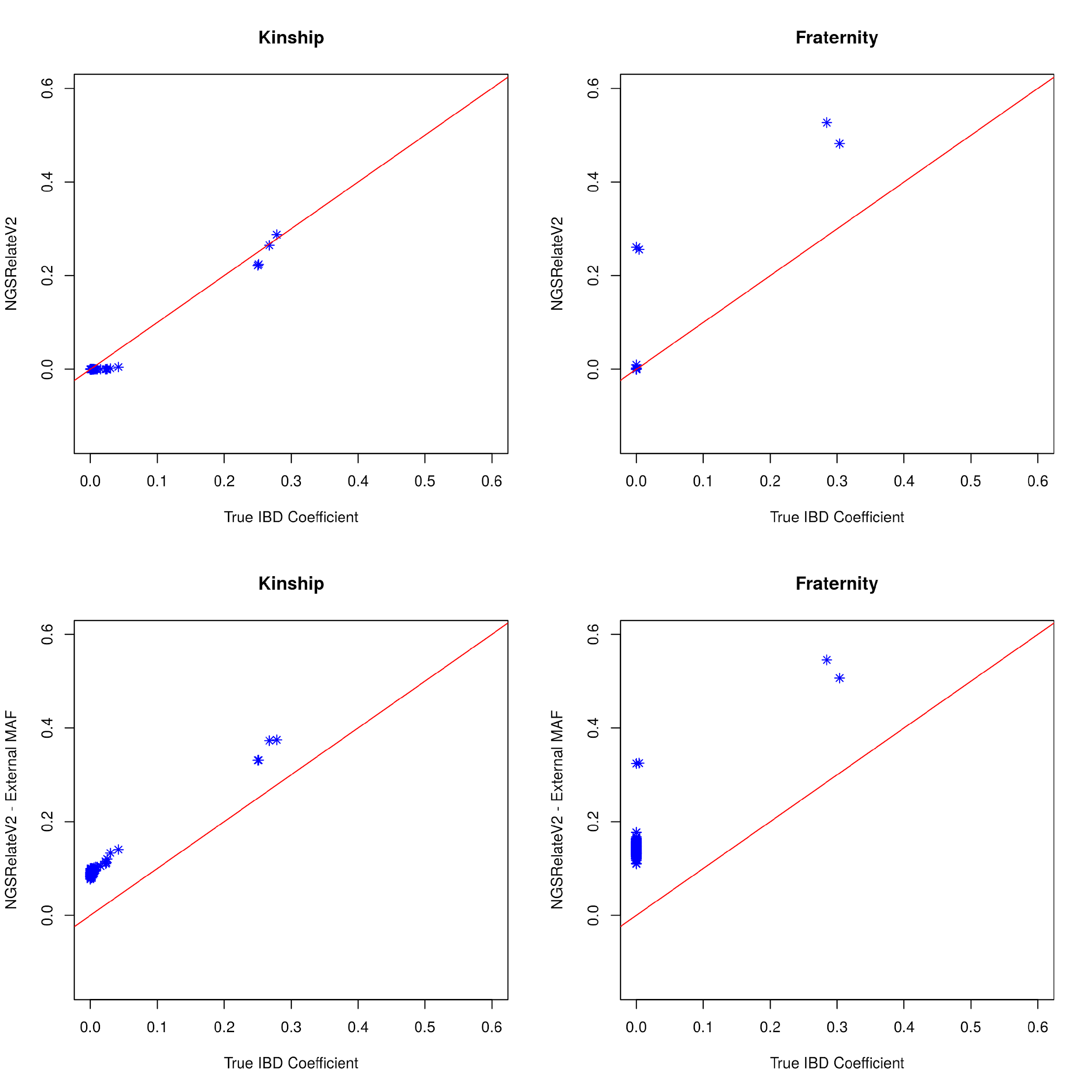


**Supplementary Figure S9**

Testing LowKi on a small sample size of only 20 individuals. In the case of a very small sample size, it is possible to supply LowKi with external allele-frequencies. 20 individuals with a sequencing depth of 2× for 200,000 SNPs were simulated using Mozza and the European haplotypes of the 1000 Genomes Project. In the top two panels, LowKi is used to estimate kinship and fraternity for this small dataset; in the next two panels, LowKi is used with the e*xternalFreqs* option using allele-frequencies estimated from 100 other simulated individual created using the same simulation set up in Mozza. Similar results are presented for NGSRelateV2.

|  |  |
| --- | --- |
|  |  |
| 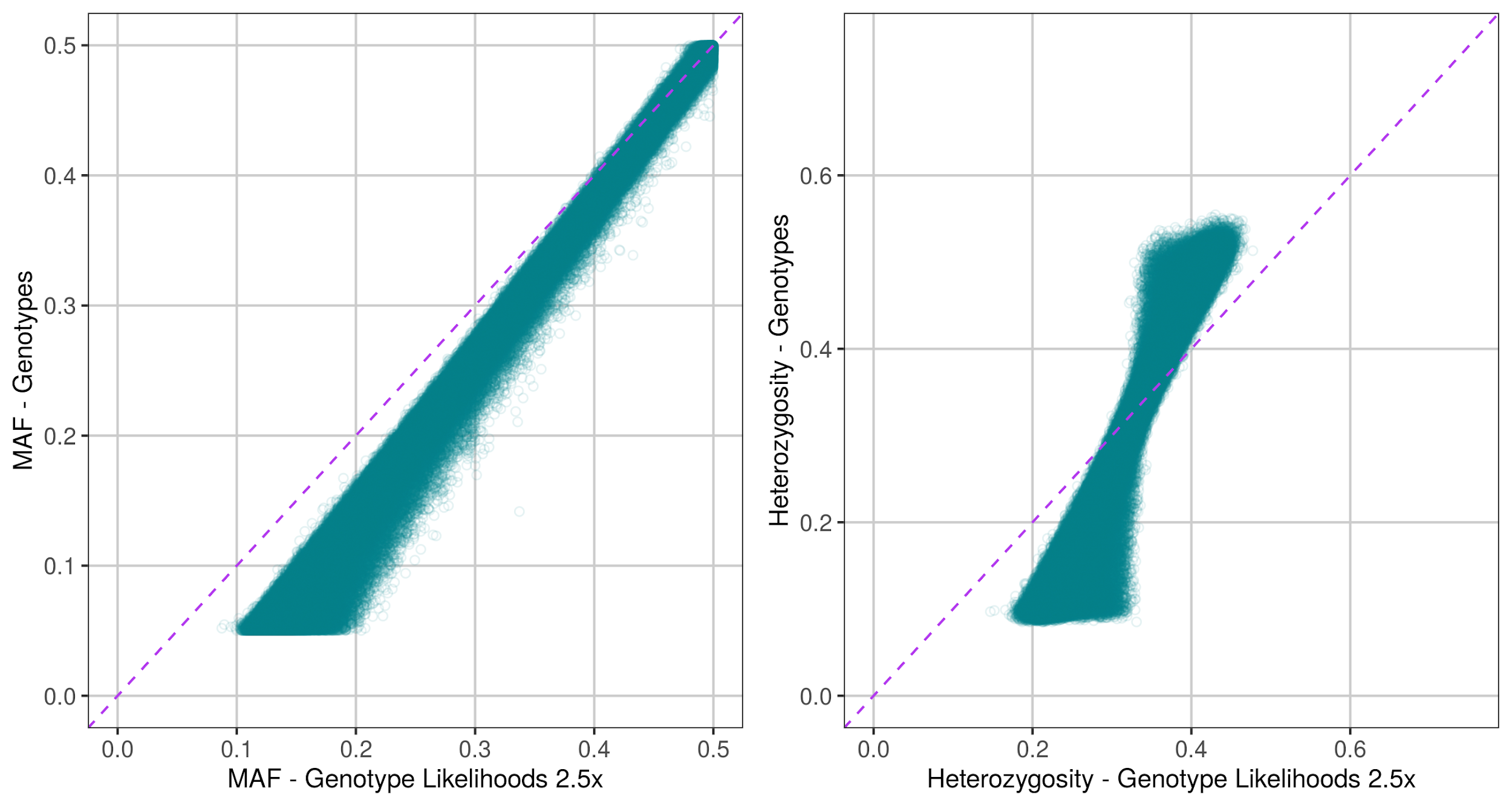 | |
| **Supplementary Figure S10**  View of how our genotype likelihood simulation affects the observed MAF and heterozygosity in the sample, going some way to explaining why correlations between individuals become biased compared to what they should be if full genotypes are observed. | |

| 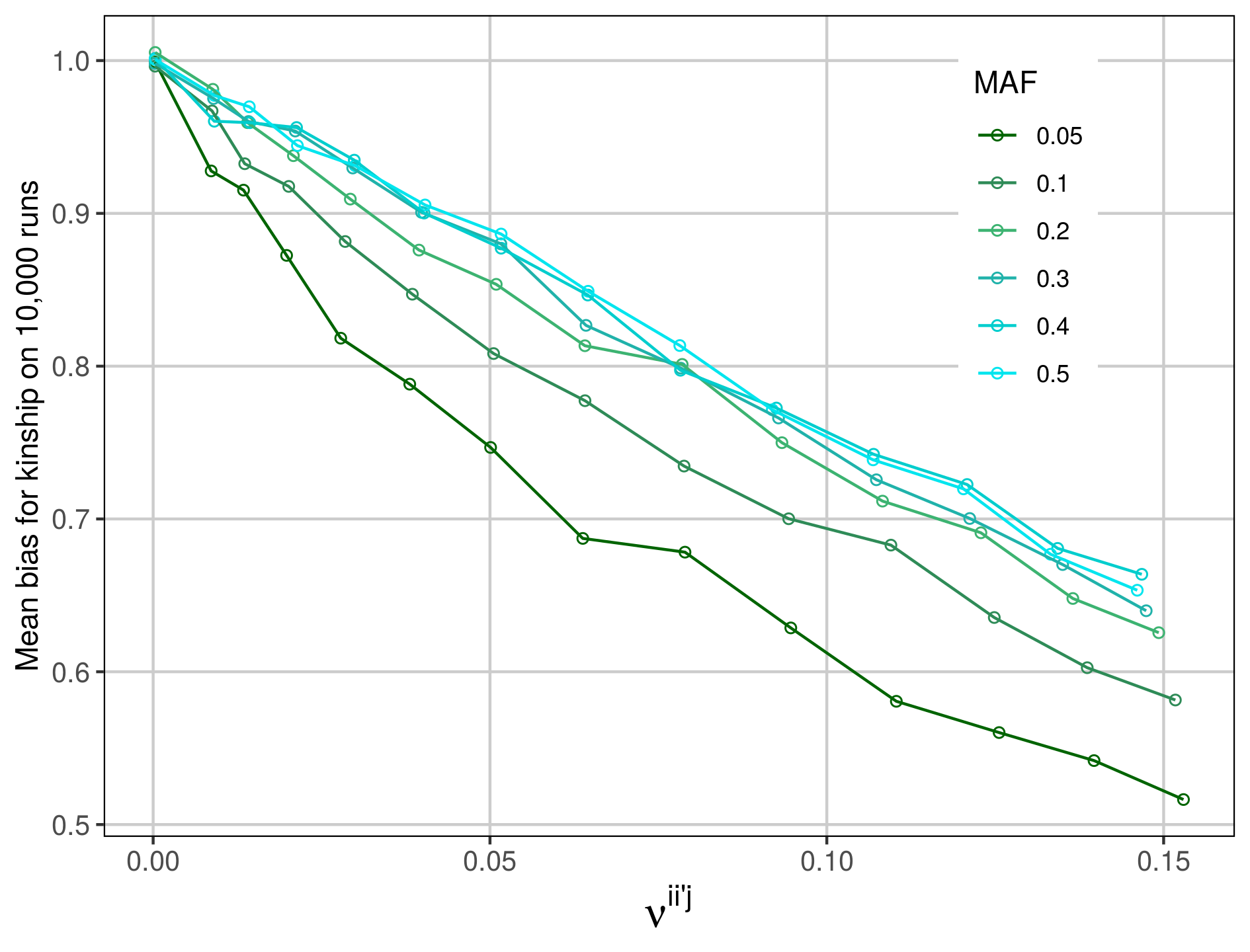 |
| --- |
| **Supplementary Figure S11**  Demonstration of the relationship between bias of kinship estimate for a sibling pair against the mean precision of genotype likelihoods ($\nu^{ii^{'}j}$ defined in main text) involved in the estimate.  Roughly linear relationships are observed, changing the Minor Allele Frequency (MAF) gives slightly different slopes. |

**Adjustment parameters**

LowKi’s adjustment procedure requires individual regression models to be fitted for pairs of individuals within the sample. All pairs can be used but in very large samples (such as CilentoSim, n=1,444) this is very time consuming and we soon observed that a subset of pairs was entirely sufficient. It was however clear that the subset of pairs selected should represent the full spectrum of possible kinship and fraternity coefficients - i.e. selecting a small set of pairs who all had a very similar ‘unadjusted estimate’ of kinship or fraternity would not give a good adjustment. Hence we have set as default that LowKi will take the 20 pairs with the lowest unadjusted estimates, 20 pairs with the highest unadjusted pairs, and then 100 further individuals are selected and all unique pairs from all individuals so far considered are used to complete the adjustment procedure. These numbers can be user defined with the option *adjust.par. T*he default setting would correspond from a setting of *adjust.par=c(20,20,100)* where the first entry represents the number of pairs with very low unadjusted estimates, the second for the very high unadjusted estimates and the third for the additional random individuals. Note that the adjustment can be skipped entirely by setting *adjust=FALSE* in LowKi. In Supplementary Figure S12 (below) the consequence of different choices of *adjust.par* is given for the estimation of fraternity from 100 individuals with 200,000 SNPs that were simulated with Mozza (https://github.com/genostats/Mozza) at a depth of 5×.


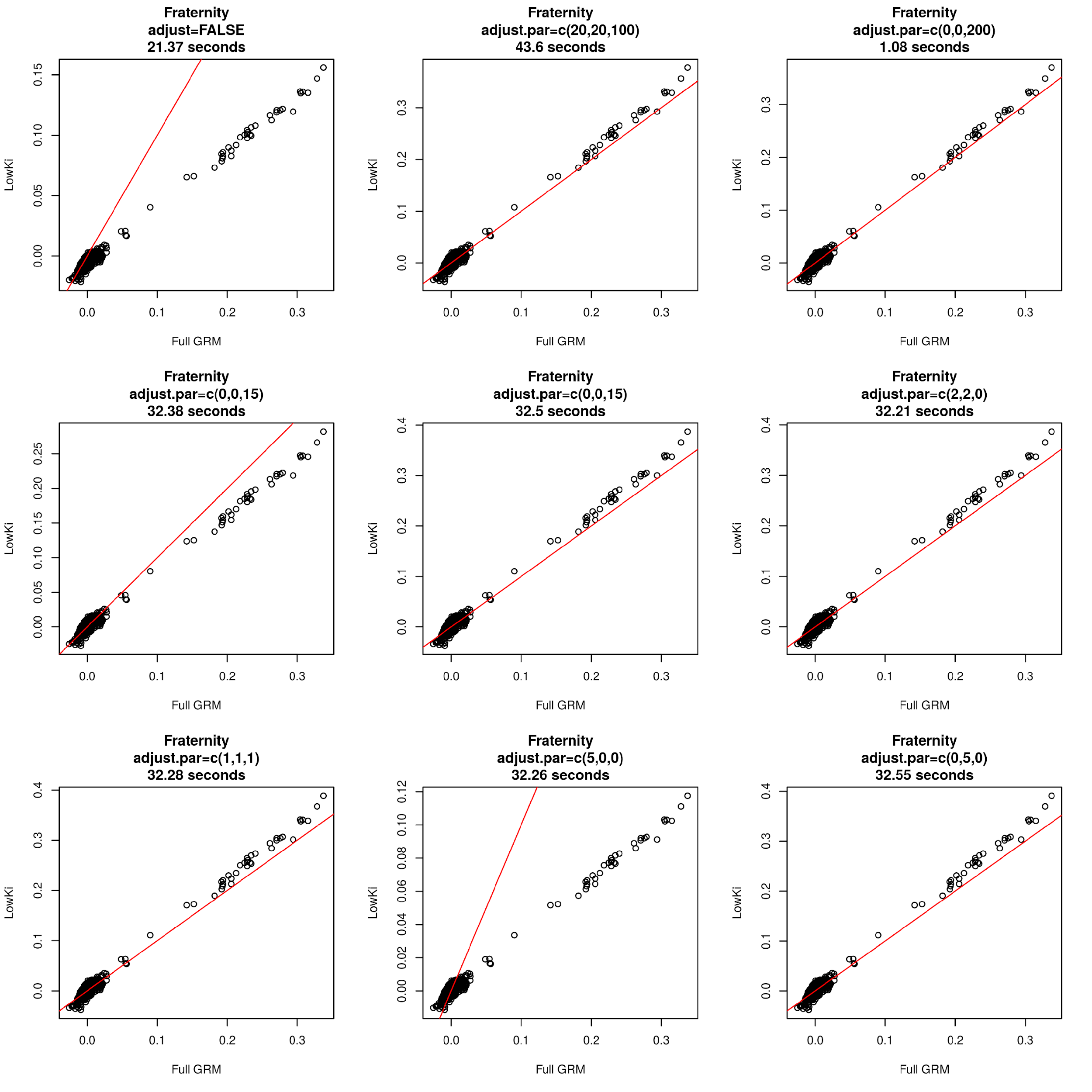


**Supplementary Figure S12**

The results of different adjustment parameters for an example of estimating fraternity among 200 individuals with 200,000 simulated SNPs at a depth of 5× based on haplotypes from the European populations of the 1000 genomes project. The sample includes a small number of related individuals by design. In the first panel the adjustment procedure is not applied; in the 2^nd^ panel the default parameters are used, in the third panel, as *adjust.par=c(0,0,200)* and that 200 individuals are present in the sample, this results in all pairs being used for the adjustment procedure at the cost of a slightly higher run time without a significant gain in performance. Whenever the user wishes to use all pairs, they should set *adjust.par=c(0,0,n)* where n is their sample size. In the 4^th^ and 5^th^ panels *adjust.par=c(0,0,15)* is used with two different random seeds with varying success; demonstrating the importance of taking some extreme pairs as indeed *adjust.par=c(2,2,0)* or *adjust.par=c(1,1,1)* )panels 6 and 7) both perform well. Using only the pairs with the lowest unadjusted estimates (panel 8, *adjust.par=c(5,0,0))* is clearly not an advisable choice; only the highest pairs (panel 9, *adjust.par=c(0,5,0))* is successful in this example but should probably also not be used.

To avoid the possibility of bad choices that could lead to spurious estimates we have restricted the user choice so that there will always be at the very least **5 pairs with the lowest unadjusted estimates and 5 pairs with the highest unadjusted estimates.** Thus choices such as *adjust.par=c(5,0,0)* and *adjust.par=c(0,5,0)* are in fact prohibited to protect the user.
